# Inosine monophosphate dehydrogenase 2 (IMPDH2) modulates response to therapy and chemo-resistance in triple negative breast cancer

**DOI:** 10.1038/s41598-024-85094-5

**Published:** 2025-01-07

**Authors:** Tatiane da Silva Fernandes, Bryan M. Gillard, Tao Dai, Jeffrey C. Martin, Kanita A. Chaudhry, Scott M. Dugas, Alyssa A. Fisher, Pia Sharma, RongRong Wu, Kristopher M. Attwood, Subhamoy Dasgupta, Kazuaki Takabe, Spencer R. Rosario, Anna Bianchi-Smiraglia

**Affiliations:** 1 Department of Cell Stress Biology, Roswell Park Comprehensive Cancer Center, CGP L3-317, Buffalo, NY 14263 USA; 2 Department of Pharmacology and Therapeutics, Roswell Park Comprehensive Cancer Center, Buffalo, NY USA; 3 Department of Breast Surgery, Roswell Park Comprehensive Cancer Center, Buffalo, NY USA; 4 Department of Biostatistics and Bioinformatics, Roswell Park Comprehensive Cancer Center, RSC R-410, Buffalo, NY 14263 USA

**Keywords:** TNBC, Chemo-resistance, Doxorubicin, Paclitaxel, IMPDH2, GTP metabolism, Ribavirin, Breast cancer, Cancer metabolism

## Abstract

Triple negative breast cancer (TNBC) is one of the deadliest subtypes of breast cancer, whose high frequency of relapse is often due to resistance to chemotherapy. Here, we identify inosine monophosphate dehydrogenase 2 (IMPDH2) as a contributor to doxorubicin resistance, in multiple TNBC models. Analysis of publicly available datasets reveals elevated IMPDH2 expression to associate with worse overall TNBC prognosis in the clinic, including lower recurrence-free survival post adjuvant/neoadjuvant therapy. Importantly, both genetic depletion and pharmacological inhibition of IMPDH2 leads to reduction of pro-tumorigenic phenotypes in multiple doxorubicin-resistant TNBC models, both in vitro and in vivo. Overall, we propose IMPDH2 as a novel vulnerability that could be leveraged therapeutically to suppress and/or prevent the growth of chemo-resistant lesions.

## Introduction

Triple negative breast cancer (TNBC) is characterized by the lack of expression of estrogen and progesterone receptors and lack of HER2 amplification, and is one of the most aggressive subtypes of breast cancer^1^. Patients with this subtype do not benefit from hormonal treatments and rely mostly on surgery, radiation, and a combination of chemotherapy treatments, including anthracyclines, taxanes and cyclophosphamide^2^. Although initial response to chemotherapy is around 40%, most patients relapse, mainly due to chemotherapy resistance^3^. At the time of relapse, the majority of patients present with metastatic disease, predominantly in the brain, bones, and lungs, resulting in a dismal 5-year survival rate of approximately 12%. Although several molecular mechanisms contributing to resistance have been identified, few are currently targetable with minimal adverse effects to the patient.

Among many molecular pathways associated with chemo-resistance, metabolic reprogramming plays an important role as cancer cells dysregulate a plethora of metabolic pathways to support their increased growth and proliferation and promote a more aggressive phenotype^4,5^. In particular, several studies have reported aberrant nucleotide metabolism, resulting from alterations to oncogenes such as myc^6,7^ and mutant p53^8^, contributing not only to tumor growth but also invasion and metastasis^9–11^. In the last few years, GTP metabolism has been highlighted as a major contributor to tumor progression in different systems, including TNBC^8–14^.

Inosine 5′-Monophosphate Dehydrogenase 1 and 2 (IMPDH1/2) catalyze the rate-limiting step in the de novo synthesis of GTP^15,16^ by oxidizing inosine monophosphate (IMP) to xanthine monophosphate (XMP) in a NAD^+^-dependent reaction^16–19^. IMPDH2 expression has been found to be vastly dysregulated in several cancer types^14,20,21^, promoting pro-tumorigenic phenotypes and leading to an elevated IMPDH2/IMPDH1 ratio^14^, while the role of IMPDH1 in cancer is less characterized. Additionally, high IMPDH2 expression has been associated with decreased sensitivity to chemotherapy in osteosarcoma^22,23^, glioblastoma^24^, and more recently colorectal cancer^25^; however, whether IMPDH2 plays a role in chemotherapy resistance in TNBC remains incompletely characterized.

A few IMPDH2 inhibitors are currently FDA-approved, although not for the treatment of cancer; for example, ribavirin is used for the treatment of viral infections^20,26^ while mycophenolate mofetil is used as an immunosuppressant post organ transplant^20,26^. The interest in repurposing IMPDH inhibitors as potential anti-cancer agents has increased over the years, with several studies highlighting the possibility of IMPDH inhibition aiding in the targeting of cancer cells, including in acute myeloid leukemia^27^, glioblastoma^28^, melanoma^9^ and small cell lung cancer^13^.

In the current study we identify IMPDH2 as an important player in the modulation of chemotherapy sensitivity of TNBC. Patient-derived data highlight a negative association between *IMPDH2* expression levels and clinical outcome, as well as induction of *IMPDH2* in response to chemotherapy. The latter was confirmed in vitro in an acute treatment setting, and with two different models of acquired resistance. Furthermore, we show that genetic or pharmacological inhibition of IMPDH2 attenuates the aggressive phenotype of different doxorubicin resistant models, both in vitro and in vivo. Overall, we identify IMPDH2 as a key contributor to chemo-resistance and as such, IMPDH2 may represent a novel therapeutic target for chemo-resistant TNBC.

## Results

### Higher IMPDH2 levels correlates with worse overall prognosis in the clinic

**Fig. 1:**
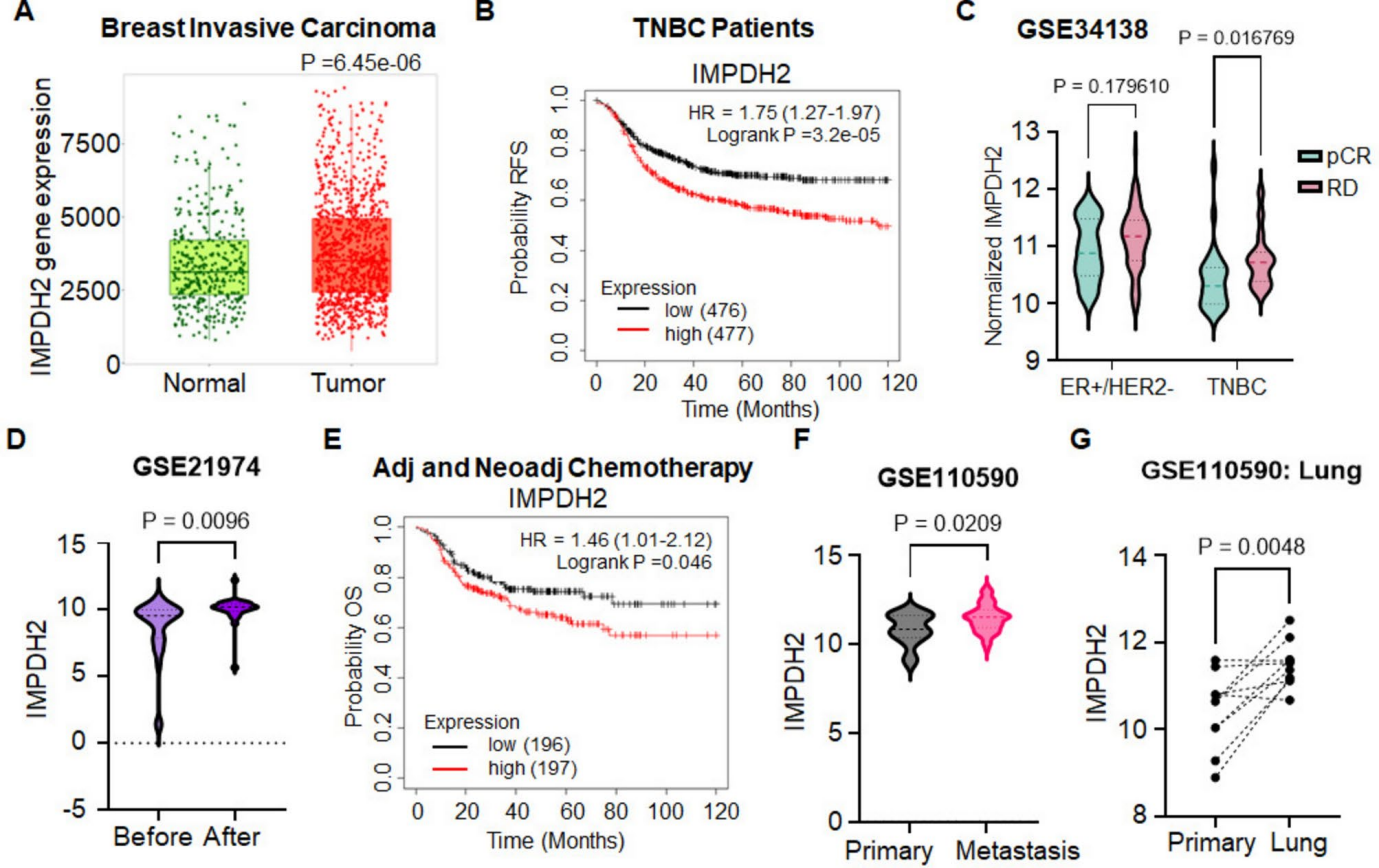
High IMPDH2 levels are associated with worse prognosis in the clinic. (**A**) IMPDH2 mRNA levels in normal vs breast invasive carcinoma tissue (KM plot database). (**B**) Relapse-free survival (RFS) of TNBC patients stratified by median *IMPDH2* mRNA levels, StGAllen dataset, n = 476/group (KM plot database). Statistics by LogRank Test. (**C**) *IMPDH2* mRNA levels in ER + /Her2- and TNBC patients that achieved pathological complete response (pCR, ER + /Her2- n = 14; TNBC n = 24) vs patients that experienced residual disease (RD, ER + /Her2- n = 108; TNBC n = 32). Data from GSE34138^29^. Statistics by Multiple Mann–Whitney Tests. (**D**) *IMPDH2* mRNA levels of primary invasive breast cancer patients before (n = 32) vs after 4 cycles of neoadjuvant chemotherapy (n = 25). Data from GSE21974^30^. Statistics by Wilcoxon Test. (**E**) RFS of TNBC patients post adjuvant/neoadjuvant chemotherapy stratified by median *IMPDH2* mRNA levels, StGAllen dataset, n = 196/group (KM plot database). Statistics by LogRank Test. (**F**) *IMPDH2* mRNA levels in primary vs multiple metastasis in 16 breast cancer patients. Data from GSE110590^31^. Statistics by Mann–Whitney Test. (**G**) IMPDH2 mRNA levels in primary vs matched lung metastasis of 7 TNBC patients. Data from GSE110590^31^. Statistics by Mann–Whitney Test.

To first investigate the relationship between IMPDH expression and breast cancer survival, we analyzed publicly available datasets. Data obtained from KM Plot (www.kmplot.com) showed that the expression of both IMPDH1 and IMPDH2 was elevated in Breast Invasive Carcinoma Tumor samples compared to Normal tissue (Fig. 1A, Suppl. Fig. S1A). While Kaplan–Meier curves generated at KM Plot showed that expression levels of neither gene could segregate the survival of bulk breast cancer patients (Suppl. Fig. S1B), individual stratification into the major PAM50 subtypes showed a significant negative association between *IMPDH2* levels and the survival of TNBC patients (Fig. 1B) but not of other breast cancer subtypes (Suppl. Fig. S1C). Similar results were obtained for *IMPDH2* and the overall survival of TNBC patients with a second dataset (Metabric, Suppl. Fig. S1D). In contrast, we found that there was no significant relationship between *IMPDH1* levels and TNBC patients’ survival (Suppl. Fig. S1E).

Analysis of data from GSE34138, which is comprised of patients that were treated with dose dense Adriamycin + Cyclophosphamide, AC^29^, revealed that TNBC patients that achieved complete pathological response (pCR) after therapy had significantly lower levels of IMPDH2 as compared to those that remained with residual disease (RD) (Fig. 1C); conversely, pre-treatment IMPDH2 levels were non-predictive for HR-positive breast cancer patients (Fig. 1C). Moreover, analysis of IMPDH2 levels in a cohort of patients before and after receiving EC + T (Epirubicin + Cyclophosphamide + Taxanes) treatment revealed that the levels of IMPDH2 in patients were significantly increased following therapy (Fig. 1D, GSE21974^30^). Consistently, *IMPDH2* levels could discriminate the survival of adjuvant and neoadjuvant chemotherapy-treated TNBC patients (Fig. 1E), suggesting that IMPDH2 induction could be a means for cancer cells to survive/escape therapy. Finally, analysis of data from GSE110590^31^, which includes matched primary and metastatic breast cancer, revealed that metastatic lesions had higher IMPDH2 levels than their corresponding primary tumors (Fig. 1F), particularly lung metastases (Fig. 1G).

Altogether, these data suggest that IMPDH2 levels could be predictive of outcome in TNBC patients, and that chemotherapy may select for IMPDH2-high cells and/or may induce upregulation of IMPDH2 as a means to survive/escape therapy, making IMPDH2 an important therapeutic target in TNBC.

### IMPDH2 levels influence chemotherapy response in TNBC cell lines

**Fig. 2:**
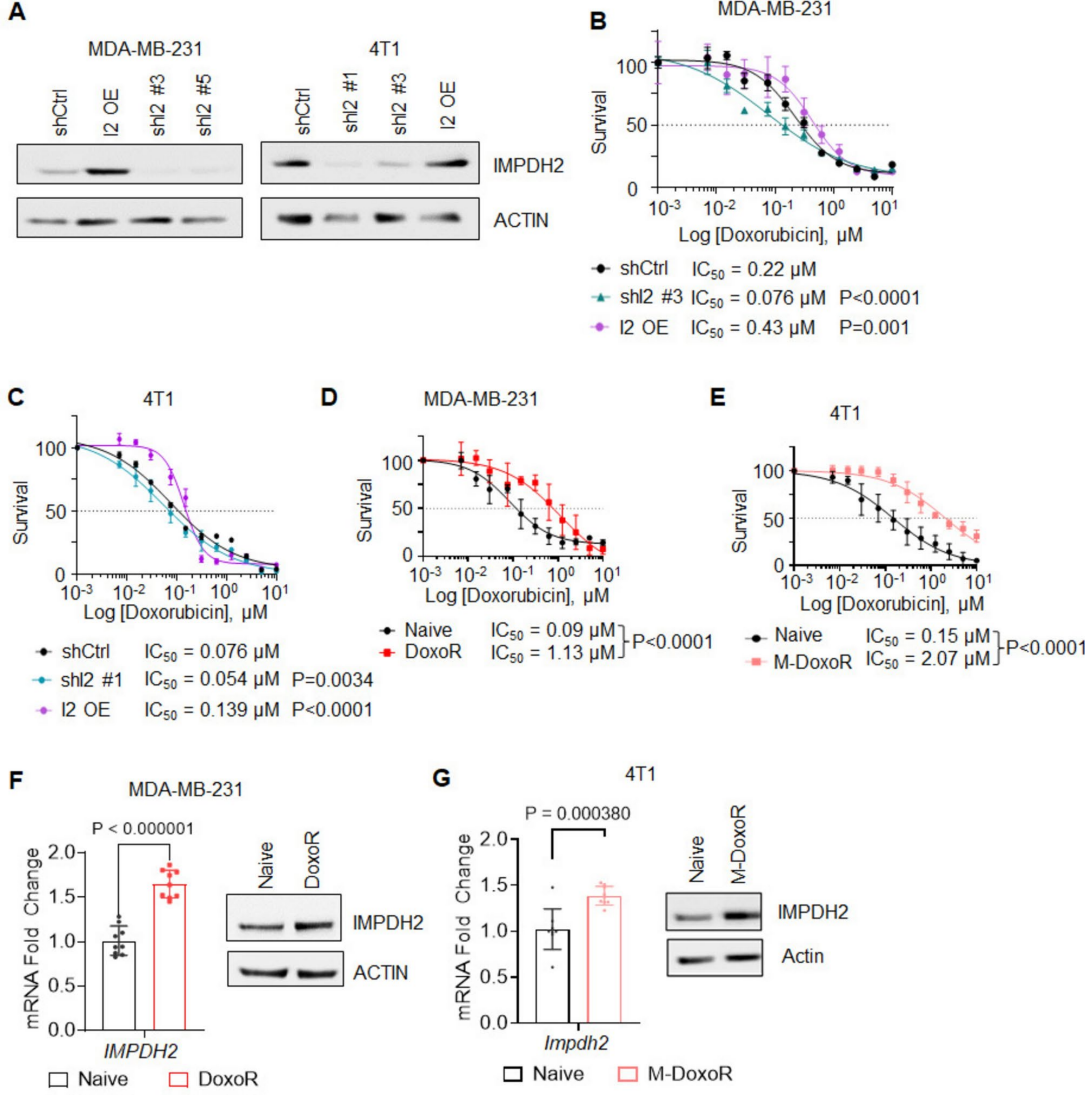
IMPDH2 levels influence response to doxorubicin. (**A**) Representative immunoblot for IMPDH2 in MDA-MB-231 (left) and 4T1 (right) cells transduced with two independent shIMPDH2 constructs (shI2), an IMPDH2 ectopic expression vector (I2OE), or their corresponding control vector. Actin is used as a loading control. Membranes were cut prior to hybridization with antibodies. Doxorubicin survival curve of MDA-MB-231 (n = 8) (**B**) or 4T1 (n = 3) (**C**) cells manipulated as in (**A**). Data is average −/+ std dev of 3 independent experiments. Doxorubicin survival curve of MDA-MB-231 naïve and DoxoR cells (**D**) or 4T1 naïve and M-DoxoR (**E**). Data is average −/+ std dev of 3 independent experiments. (**F**) Relative levels of *IMPDH2* mRNA (left) and protein (right) in MDA-MB-231 naive and DoxoR. Data is average −/+ std dev of triplicates from 3 independent experiments (**G**) Relative levels *of Impdh2* mRNA (left) and protein (right) in 4T1 naive and M-DoxoR. Data is average −/+ std dev of triplicates from 3 independent experiments. Statistics by unpaired Student’s *t-*test or by nonlinear fit regression model for IC_50_ comparisons. *p < 0.05, **p < 0.01, ***p < 0.001, ****p < 0.0001.

To investigate the relationship between IMPDH2 levels and therapy response in vitro, we used previously validated lentiviral constructs^14,32^ to transduce human MDA-MB-231 and MDA-MB-468, and mouse 4T1 TNBC cell lines to either suppress (shRNA) or ectopically express IMPDH2. We then assessed the sensitivity of cells manipulated as above to doxorubicin, one of the main chemotherapies used in the clinic for the treatment of TNBC tumors^2^. While overexpression of IMPDH2 increased resistance to doxorubicin, its depletion led to sensitization to the drug (Fig. 2A–C, Suppl. Fig. S2A–B). A similar trend was observed with paclitaxel, another commonly used chemotherapeutic agent (Suppl. Fig. S2C). The above changes were likely a direct consequence of lack of IMPDH2/GTP in the cells, as manipulation of IMPDH2 levels did not alter cell proliferation (Suppl. Fig. S2D) nor induced compensatory alterations in IMPDH1 expression levels (Suppl. Fig. S2E). Importantly, we found that acute treatment of doxorubicin led to upregulation of IMPDH2 (Suppl. Fig. S2F). Overall, these data suggest that IMPDH2 levels influence response to chemotherapeutic drugs.

### Doxorubicin resistance relies on increased GTP

To investigate the role of IMPDH2 in doxorubicin-acquired resistance, we created two doxorubicin-resistant models using human MDA-MB-231 (DoxoR) and mouse 4T1 (M-DoxoR). Cells were cultured in the presence of increasing concentrations of doxorubicin until cells proliferated normally and a significant shift in IC_50_ was observed (Fig. 2D,E). Of note, both resistant models underwent morphological changes similar to what has been reported in previous studies^33^ (Suppl. Fig. S3A–B). RT-qPCR and immunoblot analyses revealed increased levels of IMPDH2 in the resistant cells compared to their naïve counterparts (Fig. 2F,G), underscoring the potential importance of IMPDH2 in the process of acquired resistance.

**Fig. 3:**
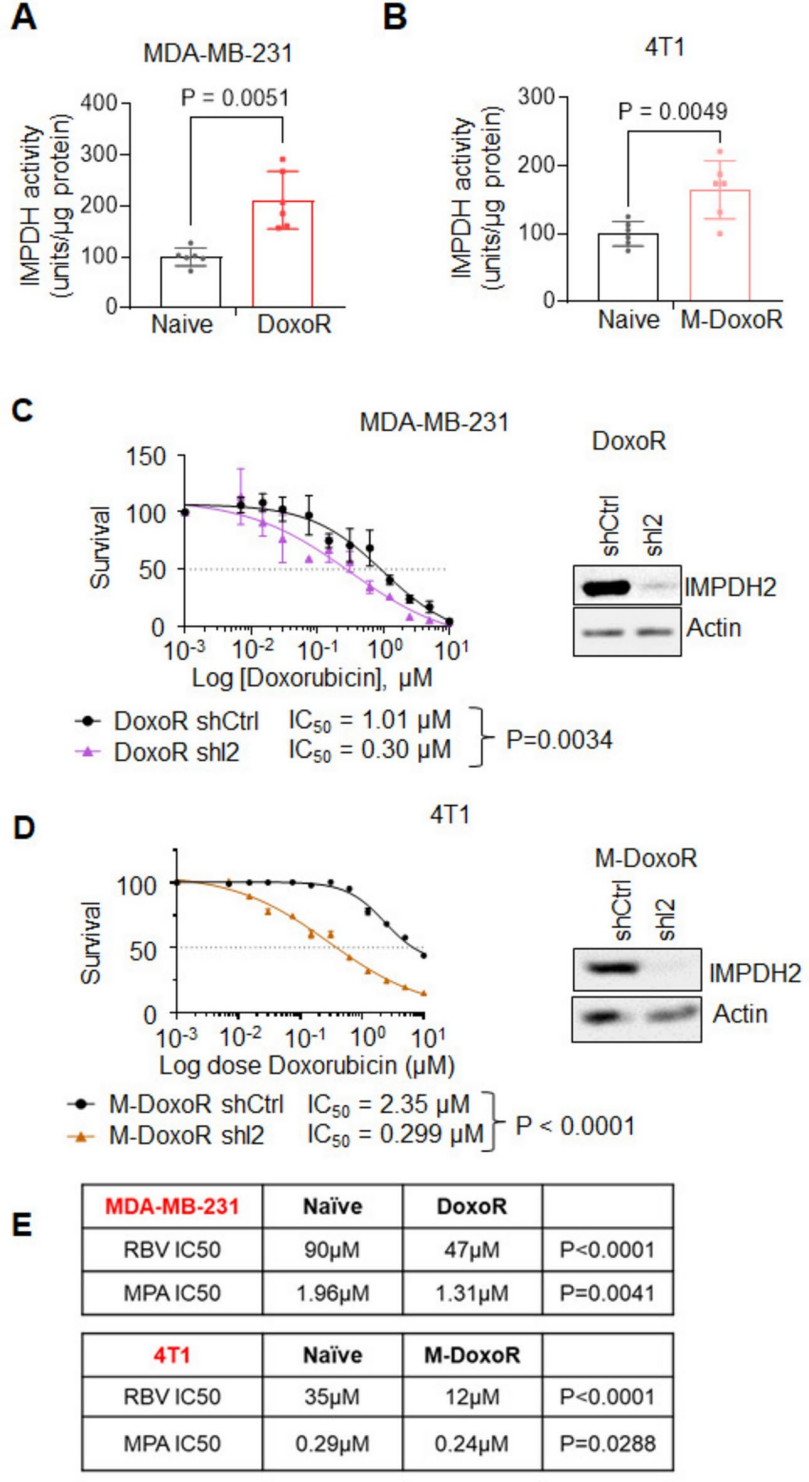
Doxorubicin resistance is accompanied by increased IMPDH2 activity and enhanced sensitivity to IMPDH2 inhibition. IMPDH activity assay in naïve and DoxoR MDA-MB-231 (**A**) or 4T1 (**B**) cells. Data is average −/+ std of 3 independent experiments. Statistics by unpaired Student’s *t*-test. (**C**) Doxorubicin survival curve (n = 3) of MDA-MB-231 DoxoR cells transduced with shIMPDH2 (shI2) or its corresponding silencing control (shCtrl). Immunoblot confirms IMPDH2 depletion; actin is used as loading control. Membranes were cut prior to hybridization with antibodies. (**D**) Doxorubicin survival curve (n = 4) of 4T1 DoxoR cells transduced with shImpdh2 (shI2) or its corresponding silencing control (shCtrl). Immunoblot confirms Impdh2 depletion; actin is used as loading control. Membranes were cut prior to hybridization with antibodies. (**E**) Ribavirin (RBV) and MPA IC_50_ in MDA-MB-231 and 4T1 naïve or doxorubicin-resistant cells (n = 4).

An IMPDH activity assay^34^ indirectly confirmed the functional activity of the elevated IMPDH2 in the resistant cells (Fig. 3A,B) and rNTPs quantification via high-performance liquid chromatography (HPLC) revealed a concomitant 20%-50% increase in intracellular GTP in both resistant models as compared to their naïve counterparts (Suppl. Fig. S4A). The marked increase in intracellular GTP could also be partially attributed to increased levels of GMPS (the enzyme directly downstream of IMPDH2 in the GTP synthesis pathway, Suppl. Fig. S4B) and reduced levels of GMPR (a functional antagonist of IMPDH2 and GMPS, Suppl. Fig. S4B) in resistant as compared to naïve cells.

To further investigate the importance of IMPDH2 in chemo-resistance, we transduced both resistant models with short hairpins against IMPDH2 as above (Fig. 3C,D). IMPDH2 depletion resulted in a significant decrease in intracellular GTP, as detected by HPLC (Suppl. Fig. S4C). At the same time, depletion of IMPDH2 restored the cells’ sensitivity to doxorubicin to levels comparable to those of their respective parental naïve lines (Fig. 3C,D).

Several metabolic enzymes have been shown to have unconventional functions that are independent of their catalytic ability^35–39^. To investigate whether IMPDH2’s role in doxorubicin resistance depends on more than its ability to stimulate GTP synthesis, we rescued IMPDH2 levels in DoxoR shIMPDH2 cells to levels similar to control with vectors encoding for either a catalytic active IMPDH2 (wild-type, I2R-WT) or a catalytic dead mutant (I2R-CD) carrying point-mutations at C133A, G326A and D364A^40^ (Suppl. Fig. S4D). These constructs were modified to be resistant (I2R) to the shRNA used for the knock-down, as previously described^32^. HPLC analysis confirmed the rescue of intracellular GTP levels to those of control in I2R-WT-but not in I2R-CD-expressing cells (Suppl. Fig. S4E). The reconstitution of DoxoR-shIMPDH2 cells with the I2R-WT construct resulted in increased resistance to doxorubicin, while the presence of I2R-CD did not alter the DoxoR-shIMPDH2 cells’ sensitivity (Suppl. Fig. S4F). These data suggest that IMPDH2’s role in modulating doxorubicin sensitivity is catalytical rather than structural and that GTP production is an essential component of resistance. This was further confirmed with additional genetic manipulation in DoxoR cells to deplete intracellular GTP levels (namely, GMPS depletion or GMPR overexpression, Suppl. Fig. S4G). Altogether, our data highlight a new vulnerability of chemo-resistant cells, centered on GTP production.

### IMPDH2 modulates pro-tumorigenic phenotypes of doxorubicin-resistant cells

To further investigate the reliance of chemo-resistant cells on IMPDH2 and GTP production, DoxoR and M-DoxoR cells were treated with the IMPDH2 inhibitors ribavirin (RBV) and mycophenolic acid (MPA), both of which are already FDA-approved, although for indications other than cancer^41–43^. Interestingly, both resistance models displayed enhanced sensitivity to IMPDH2 inhibition in comparison to their respective naive cells (Fig. 3E), suggesting that IMPDH2 inhibition could be exploited for therapeutic purposes.

**Fig. 4:**
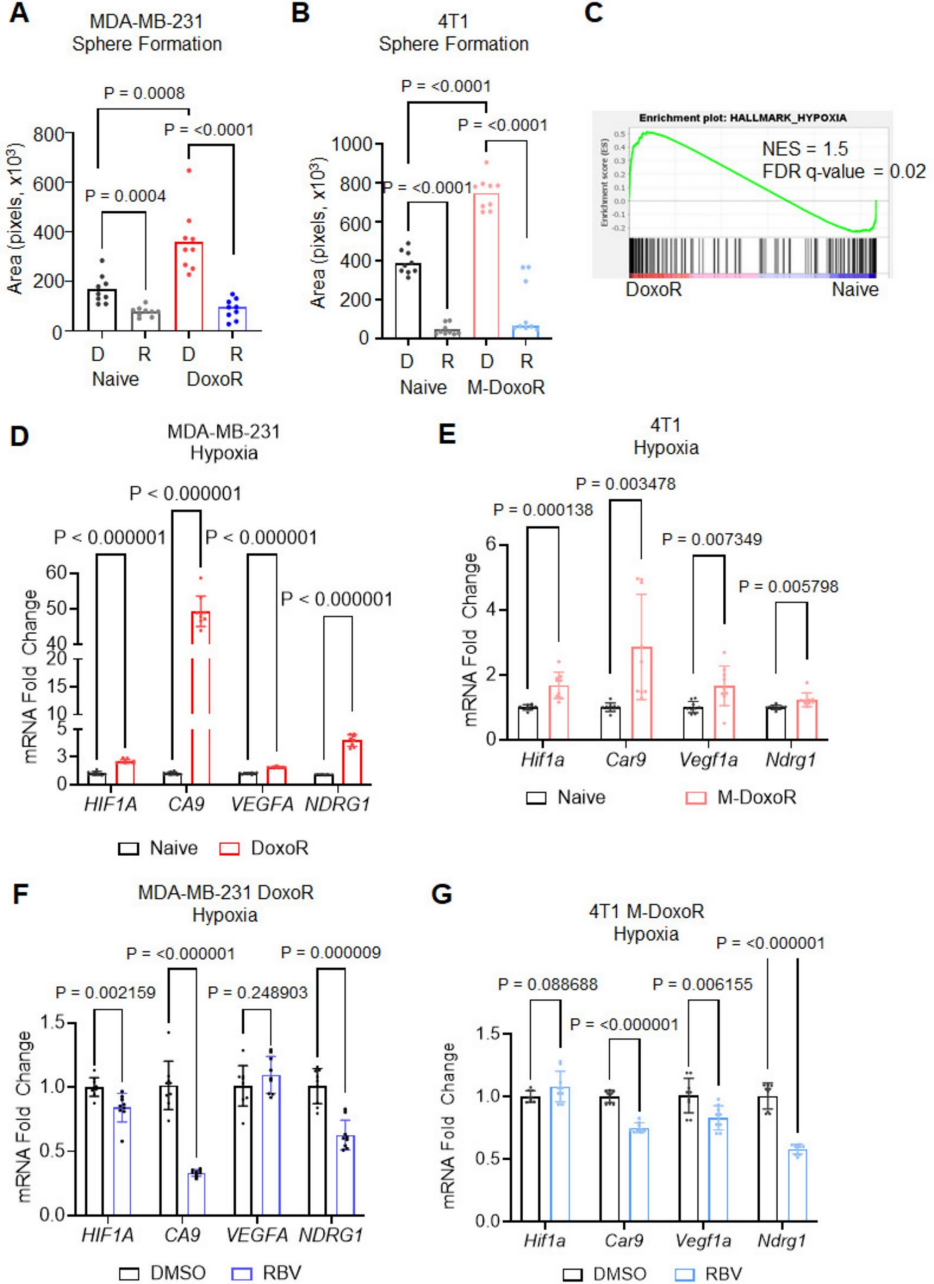
IMPDH2 inhibition suppresses increased sphere formation and hypoxic genes expression in DoxoR cells. Tumor sphere formation analysis in MDA-MB-231 (**A**) and 4T1 (**B**) naïve or doxorubicin resistant, treated with DMSO (D, vehicle control) or ribavirin (R, 25 μM). Data is average −/+ std dev of 3 independent experiments. Statistics by two-tailed Student’s t-test. (**C**) GSEA analysis for Hallmark_Hypoxia from RNA-seq of MDA-MB-231 naïve and DoxoR. RT-qPCR analysis of hypoxic genes in MDA-MB-231 naïve and DoxoR (**D**) or 4T1 naive and M-DoxoR (**E**) cells; RT-qPCR analysis of hypoxic genes in MDA-MB-231 (**F**) or 4T1 (**G**) doxorubicin-resistant cells treated with DMSO or ribavirin (50 μM MDA-MB-231; 30 μM 4T1). Data is average −/+ std dev of 3 independent experiments. Statistics by two-tailed Student’s t-test.

Resistance to chemotherapy has been shown to increase aggressive phenotypes in cancer cells^44,45^. Tumor sphere formation assays were thus carried out in MDA-MB-231 and 4T1 naïve and doxorubicin-resistant systems in the presence of ribavirin. As expected, DoxoR and M-DoxoR cells treated with vehicle control (DMSO) formed larger spheres than their respective parental cells (Fig. 4A,B, Suppl Fig. S5A). Ribavirin treatment was able to significantly reduce tumor sphere formation in both naïve and resistant settings, in both models (Fig. 4A,B, Suppl Fig. S5A). To confirm on target effects, experiments as above were performed in DoxoR or M-DoxoR cells transduced with shRNA constructs toward IMPDH2, which yielded similar results in terms sphere formation reduction (Suppl Fig. S5B,C).

As tumor cells continue to grow, they induce the formation of a hypoxic environment which further exacerbates cancer cells aggressiveness, metastatic potential, and resistance^46^. Consistently, gene set enrichment analysis (GSEA) of RNAseq data from MDA-MB-231 cells naïve and DoxoR showed an enrichment in hypoxia signature in DoxoR cells respect to naïve (Fig. 4C), which was further validated by RT-qPCR with select targets in both MDA-MB-231 and 4T1 models (Fig. 4D,E). Interestingly, IMPDH2 depletion in DoxoR cells was sufficient to cause a de-enrichment of the hypoxic signature in GSEA analysis (Suppl. Fig. S5D) and IMPDH2 pharmacological inhibition led to a decrease in markers as above in both resistance models (Fig. 4F,G).

Altogether, these results suggest IMPDH2 could be a viable target for therapy, especially in chemo-resistant settings.

### IMPDH2 inhibition reduces chemo-resistant tumor growth in vivo

**Fig. 5:**
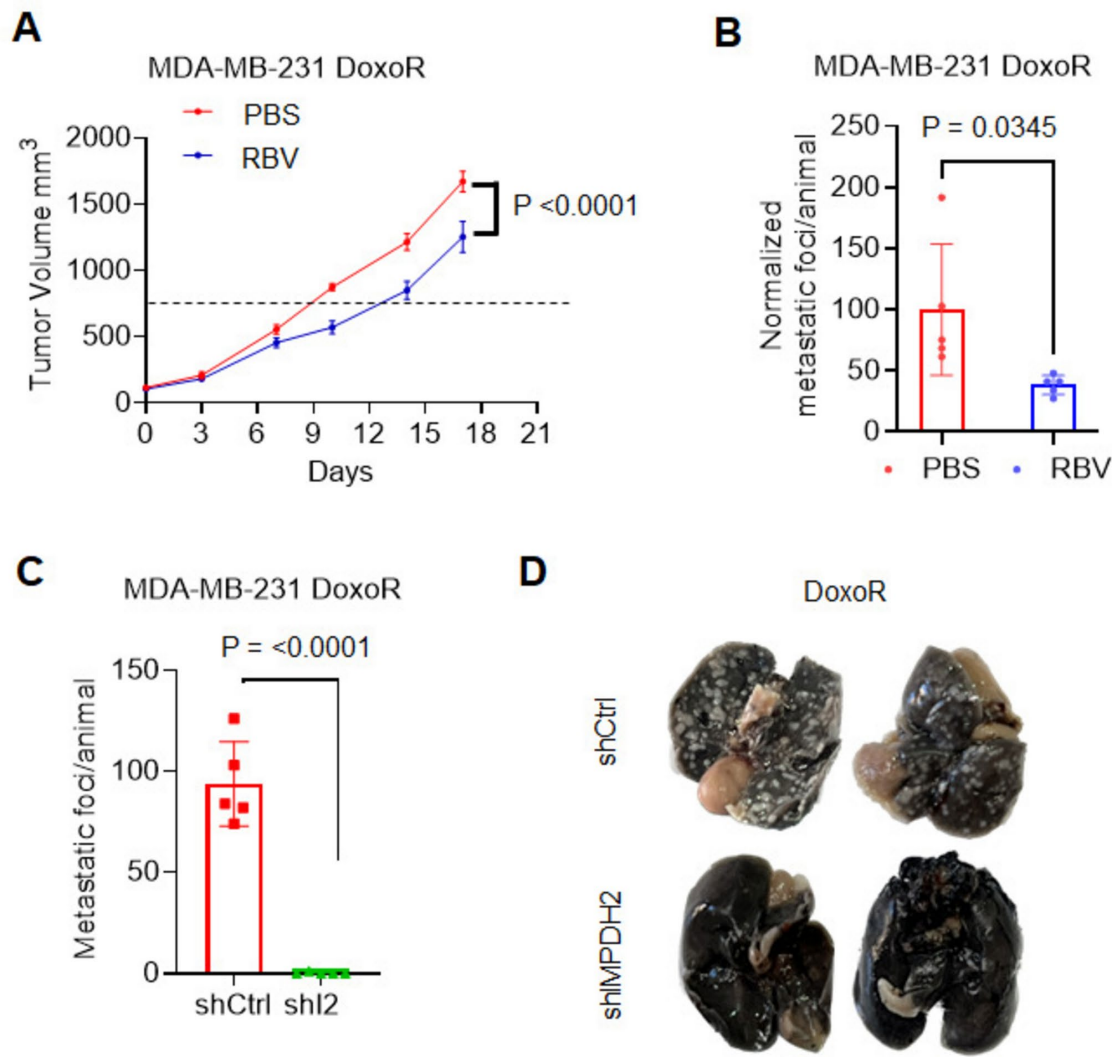
IMPDH2 inhibition reduces the tumor growth and metastatic burden of DoxoR cells in vivo. (**A**) 1 × 10^6^ MDA-MB-231 DoxoR cells were implanted orthotopically in the mammary fat pad of female NSG mice (n = 9). Treatment with PBS or ribavirin (3 mg/Kg in PBS) by i.p. injections (5 days/week) started when tumor reached 100 mm^3^ (day 0). Tumors were measured twice/week. Animals were humanely euthanized when a tumor in any group reached the limit set by IACUC protocol. Data is average −/+ SEM. Statistics by 2-way ANOVA test. (**B**) 1.5 × 10^5^ MDA-MB-231 DoxoR cells were injected in the tail vein of female NSG mice (n = 5). Treatment with PBS or ribavirin (10 mg/Kg in PBS) by i.p. injections (5 days/week) started 4 days post-injection. Animals were humanely euthanized after 3 weeks. Experimental metastases were visualized with India ink stain and quantified manually. Statistics by two-tailed Student’s *t*-test. (**C**) 1.5 × 10^5^ MDA-MB-231 DoxoR cells transduced with shIMPDH2 (shI2) or corresponding non-silencing control (shCtrl) were injected in the tail vein of female NSG mice (n = 5). Animals were humanely euthanized after 3 weeks. Experimental metastases were visualized with India ink stain and quantified manually. Statistics by two-tailed Student’s *t*-test. (**D**) Representative images of India ink-stained lungs from (**C**).

To investigate the effects of IMPDH2 pharmacological inhibition in chemo-resistant cells in vivo, MDA-MB-231 DoxoR cells were implanted orthotopically in the mammary fat pad of NSG female mice. Once tumors reached 100 mm^3^, mice were randomized into two treatment groups (n = 9/group) and treated daily (5 days/week) with i.p. injections of ribavirin (3 mg/Kg) or vehicle control. IMPDH2 inhibition was sufficient to reduce the growth of the tumors (Fig. 5A). Likewise, ribavirin treatment reduced the growth of experimental metastases of DoxoR cells. Cells were injected in the tail vein and allowed to start colonizing the lungs for 4 days, at which point mice were randomized into treatment groups as above for 3 weeks. A 3 mg/Kg dose of ribavirin was able to reduce, albeit not significantly, the number of lung nodules (Suppl. Fig. S6A, n = 3/group); however, at 10 mg/Kg ribavirin significantly reduced the growth of metastatic lesions as compared to vehicle control (Fig. 5B, n = 5/group). Importantly, genetic depletion of IMPDH2 completely abrogated the growth of lung metastases, despite the cells’ ability to reach the lungs, as evidenced by the presence of luciferase activity in cells transduced with a luciferase expression vector prior to injection (Fig. 5C,D and Suppl. Figure S6B).

**Fig. 6:**
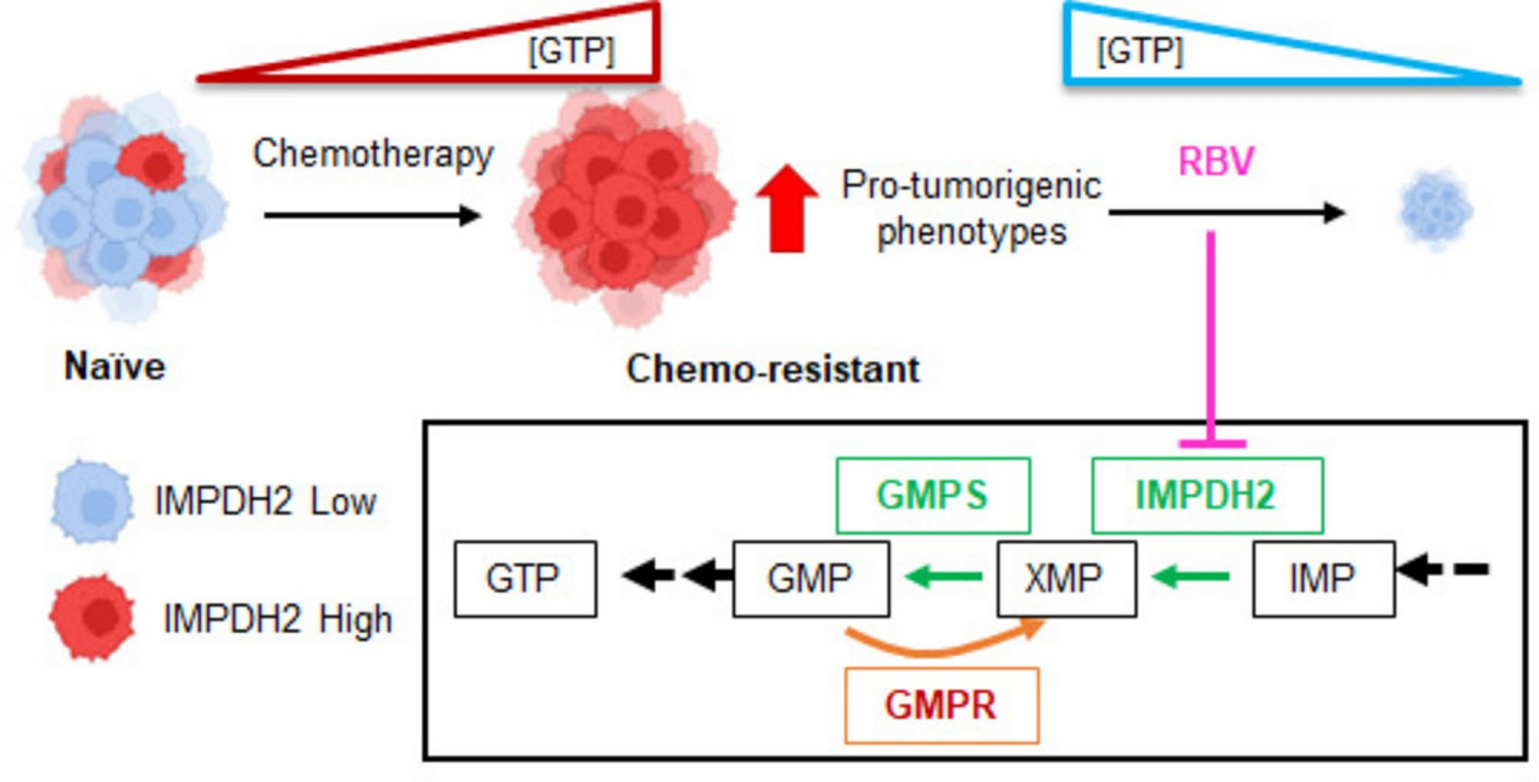
Working model. Chemotherapy treatment of TNBC selects for cells with high or induced IMPDH2 expression. The resulting chemo-resistant cells possess high GTP levels and increased pro-tumorigenic phenotypes but are exquisitely sensitive to iMPDH2 inhibition. Figure partly created in BioRender (https://BioRender.com Bianchi-Smiraglia, A. (2025) https://BioRender.com/q21s162).

Altogether, these data strongly suggest that IMPDH2 inhibition could be an important therapeutic target to reduce the growth of primary and metastatic chemo-resistant lesions (Fig. 6).

## Discussion

Chemo-resistance, being considered one of the major contributors to cancer related death, remains a great challenge in cancer treatment. While many signaling pathways have been associated with the establishment of resistance, few are currently therapeutically targetable without causing major toxicities. Our findings reveal that the metabolic enzyme IMPDH2 is a novel contributor to doxorubicin resistance in TNBC, and that its pharmacological inhibition can reduce the growth of chemo-resistant lesions.

High IMPDH2 expression has been previously shown to reduce sensitivity to treatment in various drug-naïve systems, such as cisplatin in osteosarcoma^22,23^, temozolomide in glioblastoma^24^, and oxaliplatin in colorectal cancer^25^. In the current study, we expand this knowledge to doxorubicin and triple negative breast cancer, both in the naïve and doxorubicin-resistant settings. Our findings show that perturbation of IMPDH2 levels affect the response of multiple TNBC cell lines of both human and mouse origin to doxorubicin and paclitaxel, two commonly used chemotherapies for this disease. Furthermore, stepwise acquisition of doxorubicin resistance resulted in increased levels of IMPDH2 and its endpoint metabolite GTP, which paradoxically created a novel vulnerability in the cancer cells, rendering them exquisitely sensitive to IMPDH2 depletion or inhibition, both in vitro and in vivo. Similar results were obtained with doxorubicin-resistance achieved in cells that survived multiple rounds of acute treatment with a high dose of the drug (not shown). Thus, our data suggest that IMPDH2 plays a role in both innate and acquired chemo-therapy resistance.

Chemo-therapy resistance has been linked, among others, to metabolic reprogramming, a known contributor to cancer aggressiveness; and nucleotide metabolism, particularly GTP, has been associated with increased invasion and progression of cancer in multiple systems^9–14^. As such, several groups have been exploring the repurposing of GTP-suppressing drugs for anti-cancer treatment^9,47,48^. Different IMPDH inhibitors have already received FDA approval for indications other than cancer, such as viral infections (ribavirin) and immune suppression post-organ transplant (mycophenolate mofetil)^20,26^, and multiple phase I-IV clinical trials have been initiated and then terminated without substantial reports (reviewed in Ref^20,26^). Thus, more comprehensive evaluations of the effects of IMPDH2 inhibition in cancer with these drugs are needed. At the same time, new and improved IMPDH2 inhibitors are being developed and characterized. Shikonin is a reversible competitive inhibitor of IMPDH that binds to IMPDH2 with higher affinity than IMPDH1^49^, and AVN-944 is a noncompetitive IMPDH inhibitor that has reached phase 1 clinical trial for advanced hematologic malignancies (NCT00273936), keeping the quest for IMPDH2 inhibitors as cancer treatment still going. Moreover, additional enzymes in the GTP biosynthetic pathways can be targeted phaamacologically^9^, thus, expanding the palette of potential targets.

Interestingly, GTP suppression has been previously shown to dampen the activity of several GTPases^8,10,11,14^ and a recent work linked GTP-mediated activation of the small GTPase RAC1 to the ability of cancer cells to repair DNA in response to genotoxic stress^50^. These studies thus reveal the exciting possibility that IMPDH2 inhibition may synergize with various kinds of DNA damage-centered therapies such as doxorubicin, temozolomide, and radiation. Consistently, clinical trials are currently underway in glioblastoma multiforme (NCT04477200, NCT05236036) to evaluate the combination of mycophenolate mofetil (MMF, a prodrug form of the IMPDH2 inhibitor MPA, possessing better bioavailability) with temozolomide and/or radiation^50,51^. Positive results from these studies would have profound implications for the repurposing of IMPDH2 inhibitors in cancer therapy.

Importantly, our analysis of publicly available datasets revealed that IMPDH2 levels may be able to help discriminate response to therapy, and therefore survival, of TNBC patients. Combined with the findings that chemotherapy itself can induce IMPDH2 levels in a feed-forward loop toward resistance, while at the same time introducing a new metabolic vulnerability centered on GTP, our data strongly support the need to evaluate GTP suppressing drugs as anticancer treatment more thoroughly, especially in resistance settings. Moreover, due to GTP’s established role in promotion of invasion and metastasis^8–11,14^, IMPDH2/GTP inhibition would have the added benefit of potentially reducing the formation of metastatic lesions. As such, further studies will be needed to evaluate the best positioning of GTP-suppressing drugs in the current chemotherapeutic regimen to maximize the effects on patients’ survival.

A limitation of our studies comes from the in vivo experiments being conducted primarily in immune-compromised mice. As the immune system is critical in response to therapy and its efficacy can be influenced by GTP depletion, it will be beneficial to address the effects of IMPDH2 inhibition in the context of immunocompetency. Importantly, immune checkpoint inhibitors (ICI) have been incorporated in the treatment of TNBC in both the neoadjuvant (early stage) and adjuvant (advanced stage and metastatic disease) settings; however, response rates have been very variable and just a small fraction of patients has received clinical benefits from the use of ICI. Preliminary data generated in our laboratory show an enrichment in pro-angiogenic signatures in DoxoR versus naïve cells, which can be de-enriched with IMPDH2 depletion (not shown). It is well appreciated that, in patients, high angiogenesis signatures correlate with poor immune cell infiltration and poor response to ICI^52^. Combined with our findings that IMPDH2 is associated with hypoxia-like conditions, these data raise the possibility that high IMPDH2 levels may contribute to resistance to immunotherapy. This new hypothesis will need to be thoroughly and carefully investigated in relevant systems.

## Materials and methods

### Cell lines

Human TNBC MDA-MB-231 (RRID:CVCL_0062) and MDA-MB-468 (RRID:CVCL_0419) cells and mouse TNBC 4T1 (RRID:CVCL_0125) cells were a kind gift from Dr. Andrei Bakin (Roswell Park Comprehensive Cancer Center, Buffalo, NY, USA). HEK-293T (RRID:CVCL_1926) cells were a kind gift from Dr. Irwin Gelman (Roswell Park Comprehensive Cancer Center, Buffalo, NY, USA). HEK-293T, MDA-MB-231, and MDA-MB-468 were cultured in DMEM (Invitrogen, Carlsbad, CA, USA); 4T1 were cultured in RPMI (Invitrogen). All media were supplemented with 10% fetal bovine serum (Invitrogen) and 1% antibiotic–antimycotic (Invitrogen). All cell lines were authenticated via short tandem repeat (STR) sequencing and routinely tested for mycoplasma contamination at the Roswell Park Genomics Shared Resource between November 2019 and April 2024.

### Antibodies and other reagents

Rabit monoclonal antibody to IMPDH1 (Cat. #35914, RRID:AB_3662620) was purchased from Cell Signaling (Danvers, MA, USA). Rabit polyclonal antibody to IMPDH2 (Cat. #12948-1-AP, RRID:AB_2127351) and to GMPR (Cat. #15683-1-AP, RRID:AB_2111072), and monoclonal HRP-Actin (Cat. #HRP-60008, RRID:AB_2819183) were purchased from ProteinTech (Rosemont, IL, USA). Mouse monoclonal antibody to GMPS (Cat. #376163, RRID:AB_10989583) was purchased from Santa Cuz Biotechnologies (Santa Cruz, CA, USA). Mouse monoclonal antibody to α-tubulin (Cat. #T6074, RRID:AB_477582) was purchased from Sigma-Aldrich (St. Louis, MO, USA. HRP-conjugated anti-mouse (Cat. #172-1011, RRID:AB_11125936) or anti-rabbit (Cat. #170-6515, RRID:AB_11125142) secondary antibodies were from Biorad (Hercules, CA, USA). Doxorubicin was purchased from Tocris Bioscience (Bristol, UK, Cat. #2252). Paclitaxel was purchased from Selleckchem (Huston, TX, USA, Cat. #S1150). MPA (Cat. #M3536) and ribavirin (Cat. #R9644) were purchased from Sigma-Aldrich.

### Immunoblotting

Whole cell extracts were prepared in RIPA buffer (50 mM Tris–HCl, 150 mM NaCl, 1% Triton X-100, 0.5% sodium deoxycholate, 0.1% SDS, 10% glycerol, 2 mM EDTA). Samples were resolved on polyacrylamide gels and transferred to nitrocellulose membranes (Biorad, Cat. #1620115). Membranes were incubated overnight with desired primary antibodies, followed by incubation with HRP-conjugates secondary antibodies. Signals were visualized with chemiluminescence reagents (BioRad, Cat. #1705060) and the GeneGnome XRQ NPC system (Syngene, Frederick, MD, USA, RRID:SCR_022505).

### Plasmids and infections

The pCMV-VSV-G vector (RRID:Addgene_8454) was purchased from Addgene (Cambridge, MA, USA). The pCMV-psPAX2 vector (RRID:Addgene_12260) was a kind gift from Dr. Irwin Gelman (Roswell Park Comprehensive Cancer Center, Buffalo, NY, USA). The pLvp-SV4-puro (RRID: N/A) lentiviral vector was obtained from Dr. Peter Chumakov (Cleveland Clinic, Cleveland, OH, USA). pLKO-GFP (RRID: N/A) and shRNA towards Hs IMPDH2, Hs GMPS, and Mm Impdh2 were purchased from Sigma: Hs shIMPDH2 #3: TRCN0000026591; Hs shIMPDH2 #5: TRCN00000293612; Mm shImpdh2 #1: TRCN0000041338; Mm shImpdh2 #3: TRCN0000041340; shGMPS #1 TRCN0000045942^9^. Full length human IMPDH2 and GMPR ORFs cloned into the pLvp-SV4-puro lentiviral vector were described before^11,32^. Full length mouse Impdh2 ORF was purchased from Genocopoeia (Cat. #EX-Mm44017-Lv105-B-GS). All lentiviral infections were performed as previously described^14^ in the presence of 8 μg/mL hexadimethrine bromide (Sigma).

### Doxorubicin resistance models

MDA-MB-231 DoxoR cells and 4T1 M-DoxoR cells were created by culturing cells in the presence of steadily increasing concentrations of doxorubicin over a 6-month period, until a significant shift in doxorubicin IC_50_ was observed. All experiments were performed after at least 5 passages after drug removal.

### ***IC***_***50***_*** assays***

10,000 cells per well (MDA-MB-231, MDA-MB-468) or 7,000 cells per well (4T1) were seeded in 96-well plates and treated with increasing concentrations of drugs. After 48 h, cells were fixed and stained in 0.5% methylene blue in methanol/water (75:25) and solubilized in 1% SDS in PBS. Absorbance was determined at 650 nm and 540 nm. IC_50_ values were calculated and compared with GraphPad Prism (RRID:SCR_002798) with a log(inhibitor) vs normalized response nonlinear fit regression model.

### Nucleotide quantification

Nucleotides were extracted and analyzed as previously described, with minor modifications^32^. Briefly, 4 × 10^6^ cells were collected by trypsinization, extracted with 0.4 N perchloric acid and neutralized to pH 8 with potassium hydroxide. NTPs were separated and quantified using a strong anion exchange reverse phase column (Millipore Sigma, Cat #50193-U) and a gradient high-performance liquid chromatography system (Agilent 1100, Santa Clara, CA, USA) equipped with a photodiode array detector and controlled by the Agilent ChemStation B.04.03-SP1 software (RRID:SCR_015742). Nucleotides were eluted with a linear gradient of 80% 0.005 M ammonium phosphate, pH 2.8, and 20% 0.75 M ammonium phosphate, pH 3.6, to 20% 0.005 M ammonium phosphate, pH 2.8 and 80% 0.75 M, pH 3.6 over 30 min which was then maintained for an additional 5 min. The mobile phase was then returned to starting conditions over 10 min and equilibrated for an additional 30 min before the next injection. Nucleotides were identified based on their UV absorbance spectrum and quantified at either 254 nm or 281 nm by comparison to the absorbance of a known amount of authentic standard.

### IMPDH activity assay

IMPDH activity assay was performed according to the manufacturer’s instructions and as previously described^34^. Briefly, at least 1 × 10^5^ cells per sample were collected and lysed. Samples were brought to 1 µg/µL and assayed with Control Solution or Reaction Solution at 37C for 2 h. Absorbance was measured at 492 nm and enzyme activity was measured using the formula

$$IMPDH\;activity = O.D. \times 1000 \times 70\;\upmu L/(120\;\min \times 0.5\;{\text{cm}} \times 18 \times 20\,\upmu {\text{L}}) = \Delta O.D. \times 3.24.$$

### Tumor sphere formation

Cells were seeded at 100 cells/well in 96 well ultra-low attachment plates (Corning, NY, USA, Cat. No. 7007) for 7–14 days. Pictures were taken at a Nikon Ts2FL Inverted Microscope equipped with a Nikon 5 MP Digital Insight DS-Fi3 Camera twice/week. Surface areas were measured with ImageJ (RRID:SCR_003070).

### RNAseq

Total cellular RNA was isolated using the PureLink RNA Mini kit with on-column DNase treatment (Thermo Fisher Scientific) and RNAseq analysis was carried on as previously described at the Roswell Park Comprehensive Cancer Center’s Genomics Shared Resource^53,54^. Differential expression analysis was carried out with DESeq2^55^ and differential expression rank order was used for subsequent gene set enrichment analysis (GSEA)^56^, performed using the cluster profile package in R and collections available through the Molecular Signatures Database (MSigDB, RRID:SCR_016863)^57^. Overlapping differentially expressed genes from each of the DEG lists across comparisons were calculated by hypergeometric testing. All analyses were performed using R statistical software, version 4.1.1 (RRID:SCR_001905).

### Publicly available datasets analysis

Breast cancer bulk RNA-seq datasets GSE34138, GSE21974, and GSE110590 were downloaded from the Gene Expression Omnibus (GEO, RRID:SCR_005012) database. IMPDH2 expression was determined using probes from the designated GPL associated with each GEO dataset, and all associated clinical information was downloaded with each dataset. When multiple probes existed, the expression levels were averaged. The ‘gplots’ and ‘survminer’ packages in R were utilized. Additionally, data was abstracted from R and imported into Prism GraphPad (vsn 9) to generate violin plots.

### Quantitative real time pCR

Total cellular RNA was collected using the PureLink™ RNA Mini (ThermoFisher Scientific, Waltham, MA, USA Cat. #12183025) with on-column DNase treatment (ThermoFisher Scientific, Waltham, MA, USA, Cat. #12185010), as per manufacturer’s recommendations. 1 μg of total RNA was reverse transcribed using the High-Capacity cDNA Reverse Transcription Kit (Applied Biosystems Waltham, MA, USA Cat. #4368814). Quantitative real time PCR was performed on a CFX Opus 96 Real-Time PCR System (BioRad, Hercules, CA, USA Cat. #12011319) using iTaq Universal SYBR® Green Supermix (BioRad, Hercules, CA, USA. Cat. #1725121) or SsoAdvanced Universal Probes Supermix (BioRad, Hercules, CA, USA. Cat. #17255280) with the following human/mouse site-specific primers listed in Table 1. Data were analyzed using the Bio-Rad CFX Maestro software (RRID:SCR_018064).

**Table 1:** List of primers and probes used for qRT-PCR analyses.

	FWD	REV
Human primers		
RPS20	AAGGATACCGGAAAAACACCC	TTTACGTTGCGGCTTGTTAGG
IMPDH2	TaqMan probe Hs00168418_m1	
	(ThermoFisher Scientific, Waltham, MA, USA, Cat. No. 4331182)	
GMPS	GAGTCAAAGCCTGCACAACA	TTATGGGCCTCAAAATCAGC
GMPR	GAGTGCCGTCATTGAGTGTG	TCCGTATGACCCGAAAACAT
HIF1A	TCCAAGAAGCCCTAACGTGT	TGATCGTCTGGCTGCTGTAA
CA9	AGTGCTAAGCAGCTCCA	CTCAATCACTCGCCCATTC
VEGFA	TTCAAGCCATCCTGTGTG	CCGCATAATCTGCATGGT
NDRG1	CTGTCATCCTCACCTACCATG	TGCATGTCCTCGTAGTTGAAG
Mouse primers		
Rps20	GCATGCCTACCAAGACTTT	CAATGAGTCGCTTGTGGAT
Impdh2	TGGGATCCGGCTGAAGAAAT	TGCCAGCAATCAGGTAAGGA
Gmps	AGGACCCCACGGAAAAGAAT	GTCGTTGTGGTGGGTTTTGA
Gmpr	CCGATCCAAATTCCCCGAAC	ACATCTCCTGGACATGTGCA
Hif1a	TCAGCATACAGTGGCACTCA	AAGGGAGCCATCATGTTCCA
Car9	GACCTCGTGATTCTCGGCTA	GAGAAGGCCAAACACCAAGG
Vegf1a	GCTGTAACGATGAAGCCCTG	CGCTCCAGGATTTAAACCGG
Ndrg1	AGGAGAGAGAGAGGCAGGAA	GGTTGTGGGTTTCAGTGTCC

### Animal cell lines studies

All experiments involving animals were approved by the Institutional Animal Care and Use Committee (IACUC) and carried out in accordance with IACUC guidelines and regulations. For orthotopic xenografts, MDA-MB-231 DoxoR cells (1 × 10^6^) were resuspended in 50μL of 1:1 PBS and Matrigel (Corning, NY, USA, Cat. #354230) and inoculated in the left mammary fat pad of 6–8-week-old female NOD-SCID Gamma mice (n = 10), bred and housed at the Division of Laboratory Animal Resources (Roswell Park Comprehensive Cancer Center, Buffalo, NY, USA). Tumor volumes were recorded twice/week and treatments (via i.p. injections, 5 days/week) started when tumors reached ~ 100 mm^3^. Mice were randomized in 2 treatment groups (n = 5/group): (1) PBS saline (control group) and (2) ribavirin (10 mg/Kg in PBS). Mice were humanely euthanized by CO2 asphyxiation followed by cervical dislocation when a tumor volume reached 2cm^3^ or when a tumor became ulcerated. Primary tumor volume was measured twice per week and calculated with the following formula: *volume* = 1/2 ∗ (*lengt*ℎ ∗ *widt*ℎ^2^). The growth of tumors in the treatment group was compared to that of control group. The investigator measuring tumor sizes was blinded to the treatment group allocation.

For experimental metastases assays, 1.5 × 10^5^ cells were resuspended in PBS, injected via tail vein and allowed to colonize the lungs for 4 days before the start of treatments as above. Mice were humanely euthanized by CO2 asphyxiation followed by cervical dislocation at 3 weeks post-inoculation. Lungs were stained with India ink and stored in Feketes solution. Metastatic lesions were manually counted by two independent researchers who were blinded to the treatment group allocation. No animals were excluded from the study since all animals developed palpable tumors within 2 weeks of orthotopic inoculation of cells and no animals developed significant morbidity before the end of the study. These methods are reported in accordance with ARRIVE guidelines.

### Statistics

Experiments were repeated at least three independent times (exact n is indicated in figure legends). Statistical analysis was performed using Student’s *t*-test within Prism version 9 software (GraphPad, San Diego, CA), unless otherwise noted. A two-tailed *p* value < 0.05 was considered statistically significant for analyses*.* For mouse studies, tumor growth rates were compared between treatment groups using 2-way ANOVAs test at a significance level of 0.05.

## Supplementary Information

Supplementary Information.

## Data Availability

The data that support the findings of this study are available from the corresponding authors upon reasonable request. All sequencing data reported here are available in the NCBI Gene Expression Omnibus (GEO) database under accession number GSE264197.
