# Supplementary material for: Inosine monophosphate dehydrogenase 2 (IMPDH2) modulates response to therapy and chemo-resistance in triple negative breast cancer
Source: Sci Rep. 2025 Jan 7;15:1061. doi: 10.1038/s41598-024-85094-5 (PMC11707137; doi:10.1038/s41598-024-85094-5)

Inosine Monophosphate Dehydrogenase 2 (IMPDH2) Modulates Response to Therapy and Chemo-Resistance in Triple Negative Breast Cancer.

Tatiane da Silva Fernandes[1], Bryan M. Gillard[2], Tao Dai[1], Jeffrey C. Martin[1], Kanita A. Chaudhry[1], Scott M. Dugas[1], Alyssa A. Fisher[1], Pia Sharma[3], RongRong Wu[3] Kristopher M. Attwood[4], Subhamoy Dasgupta[1], Kazuaki Takabe[3], Spencer R. Rosario[2,4*], and Anna Bianchi-Smiraglia[1*]

Supplementary Figure S1

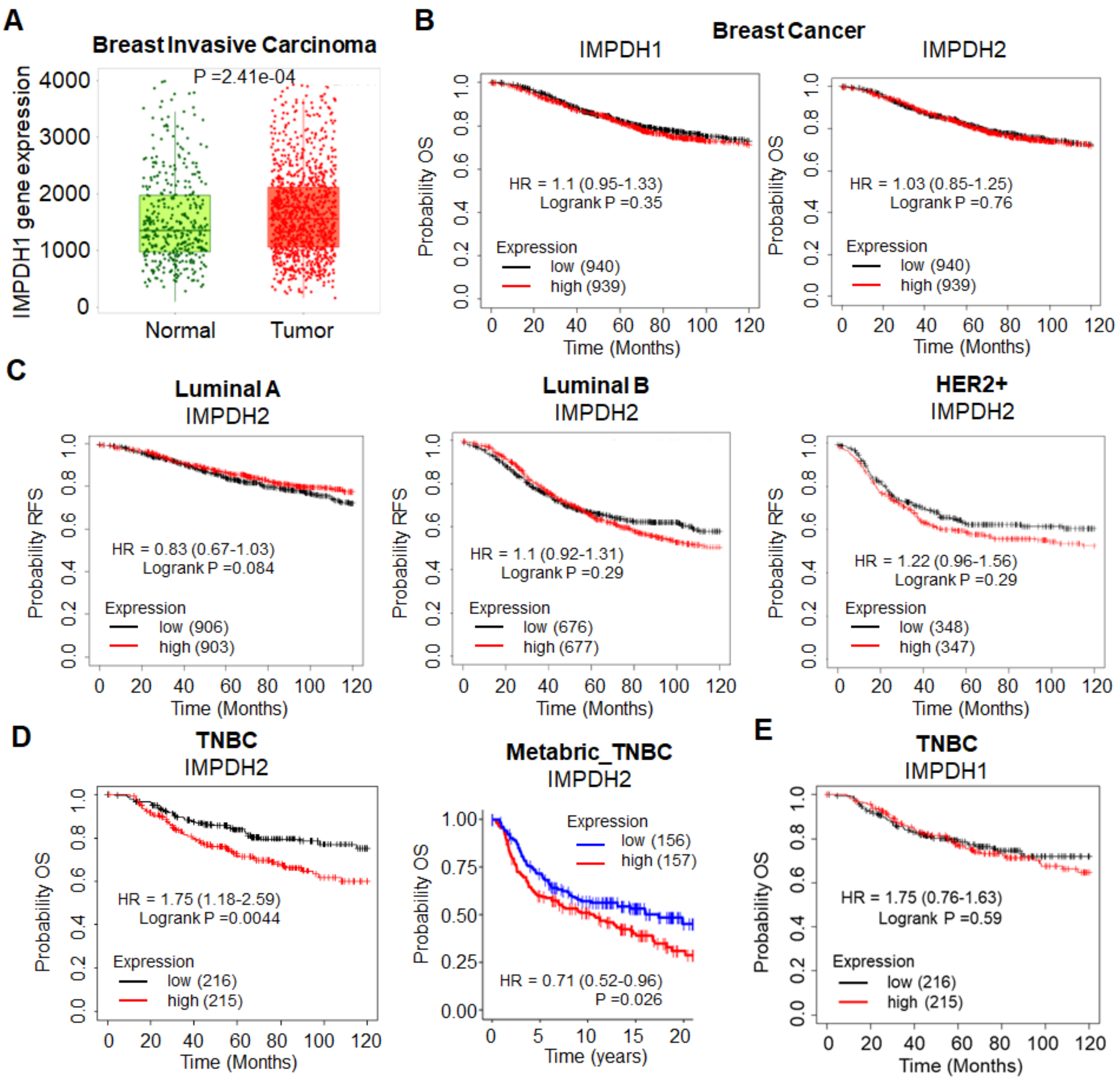

Supplemental Figure S1. IMPDH2, but not IMPDH1 levels are associates with TNBC patients' survival. (A) IMPDH1 mRNA levels in normal vs breast invasive carcinoma tissue (KM plot database). **(B)** OS of breast cancer patients (no subgrouping) stratified by median *IMPDH1* (left) or *IMPDH2* (right) mRNA levels (KM plot database). Statistics by LogRank Test. **(C)** RFS of Luminal A, Luminal B, and HER2+ patients stratified by median *IMPDH2* mRNA levels, PAM50 dataset (KM plot database). Statistics by LogRank Test. **(D)** OS of TNBC patients stratified by median *IMPDH2* mRNA level (high vs low), PAM50 dataset (left) and Metabric. Statistics by LogRank Test. **(E)** OS of TNBC patients stratified by median *IMPDH1* mRNA levels. Statistics by LogRank Test.

Supplementary Figure S2

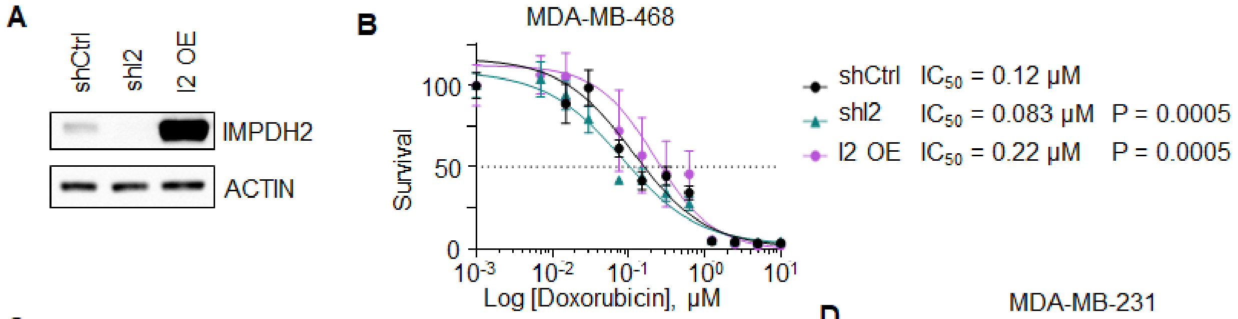

C

Paclitaxel	Ctrl IC50	shIMPDH2 IC50	
**MDA-MB-231**	0.0048μM	0.0012μM	P <0.0001
**MDA-MB-468**	0.0018μM	0.0011μM	P <0.0001

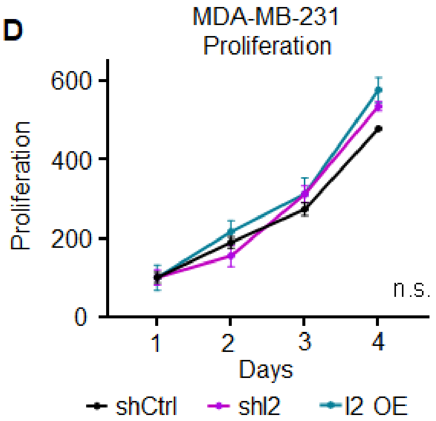

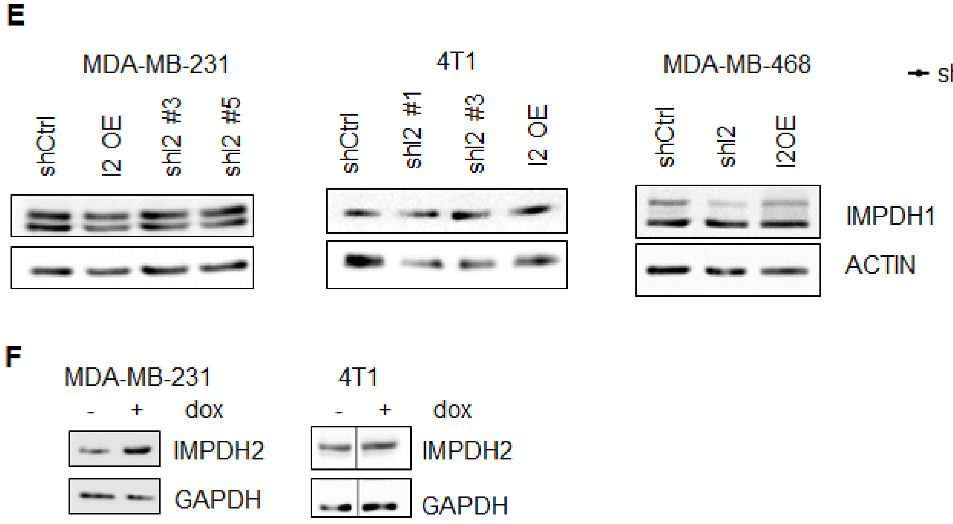

Supplemental Figure S2. Chemosensitivity depends on IMPDH2/GTP levels. (A) Representative immunoblots and **(B)** doxorubicin survival curve of MDA-MB-468 cells transduced with a shIMPDH2 constructs (shI2), an IMPDH2 ectopic expression vector (I2OE), or their corresponding control vector. Data is average -/+ std dev of 8 independent experiments. **(C)** Paclitaxel IC_{50} of MDA-MB-231 and MDA-MB-468 cells transduced with shIMPDH2 or vector control. Data is average -/+ std dev of 4 independent experiments. **(D)** Proliferation assay of MDA-MB-231 cells transduced with a shIMPDH2 constructs (shI2), an IMPDH2 ectopic expression vector (I2OE), or their corresponding control vector. Data is average -/+ std dev of 8 independent experiments. Statistics by two-tailed Student's *t*-test. **(E)** Representative immunoblot analysis of IMPDH1 levels in MDA-MB-231 (left), 4T1 (middle) or MDA-MB-468 (right) cells transduced with shIMPDH2 (shI2) or IMPDH2OE (I2OE).

Actin is used as loading control. **(F)** Representative immunoblot analysis of IMPDH2 levels in MDA-MB-231 and 4T1 treated with doxorubicin 0.15μM for 24hrs. GAPDH is used as loading control.

Supplementary Figure S3

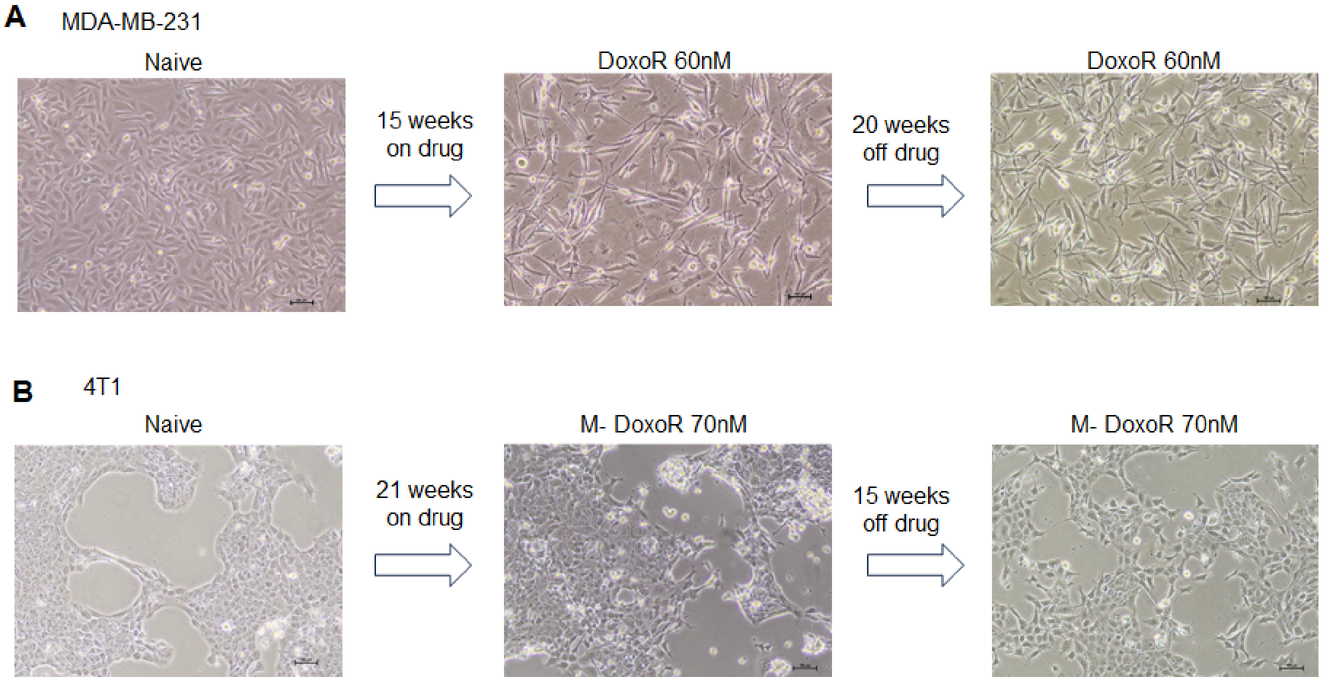

Supplemental Figure S3. Persistent morphological changes in doxorubicin-resistant cells. Representative phase contrast images of morphological changes in MDA-MB-231 **(A)** and 4T1 **(B)** before (naïve) and after the process of acquisition of resistance, which persisted for several weeks upon drug withdrawal. Scale bar, 100μm.

Supplementary Figure S4

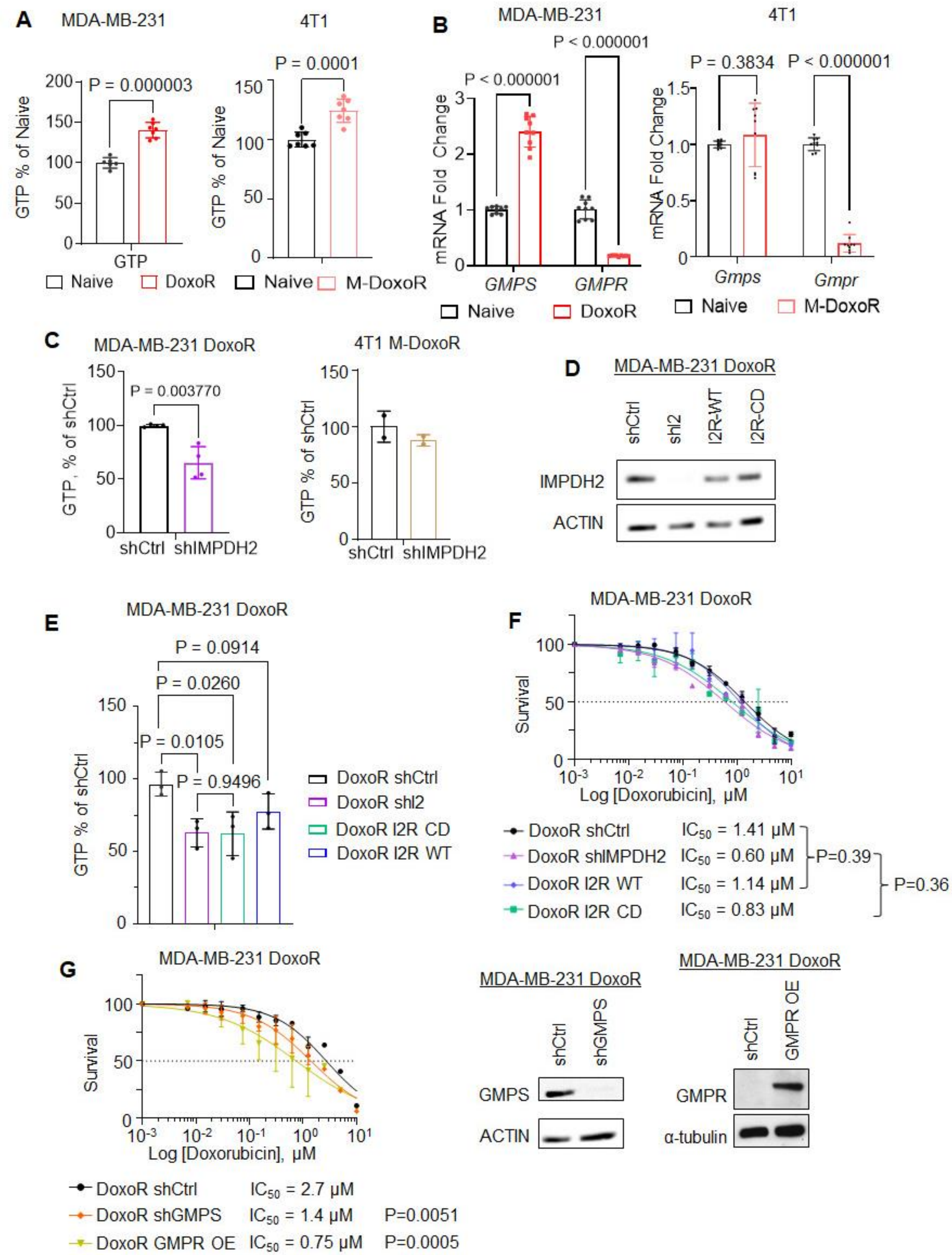

Supplemental Figure S4. Sustained GTP levels are necessary for maintenance of resistance. (A) Relative intracellular GTP levels measured by HPLC in MDA-MB-231 naive and DoxoR (right) and 4T1 naïve and M-DoxoR (left). Data is average -/+ std dev of 7 independent experiments. Statistics by unpaired Student's *t*-test. **(B)** qRT-PCR analysis for the expression levels of GTP *de novo* biosynthetic enzymes GMPS and GMPR in

MDA-MB-231 (left) or 4T1 (right) naïve and doxorubicin-resistant cells. Data is average -/+ std dev of 9 replicates from 3 independent experiments **(C)** Intracellular GTP levels quantification by HPLC in MDA-MB-231 (left) or 4T1 (right) doxorubicin-resistant cells depleted or not of IMPDH2. Data is average -/+ std dev of 2-4 independent experiments. **(D)** Representative immunoblots and **(E)** intracellular GTP levels quantification by HPLC in MDA-MB-231 DoxoR control, shIMPDH2 and shIMPDH2 rescued with wild-type (I2R-WT) or catalytic-dead (I2R-CD) IMPDH2 constructs. Data is average -/+ std dev of 3 independent experiments. **(F)** Doxorubicin survival curve of MDA-MB-231 DoxoR cells manipulated as in (D). Data is average -/+ std dev of 8 replicates from 2 independent experiments. **(G)** Doxorubicin survival curve of MDA-MB-231 DoxoR cells transduced with a previously validated shRNA against GMPS[9], a construct for ectopic expression of GMPR[11], or corresponding control vector. Data is average -/+ std dev of 8 replicates from 2 independent experiments. Immunoblots verify the intended manipulations. Actin and alpha-tubulin are used as loading controls.

Supplementary Figure S5

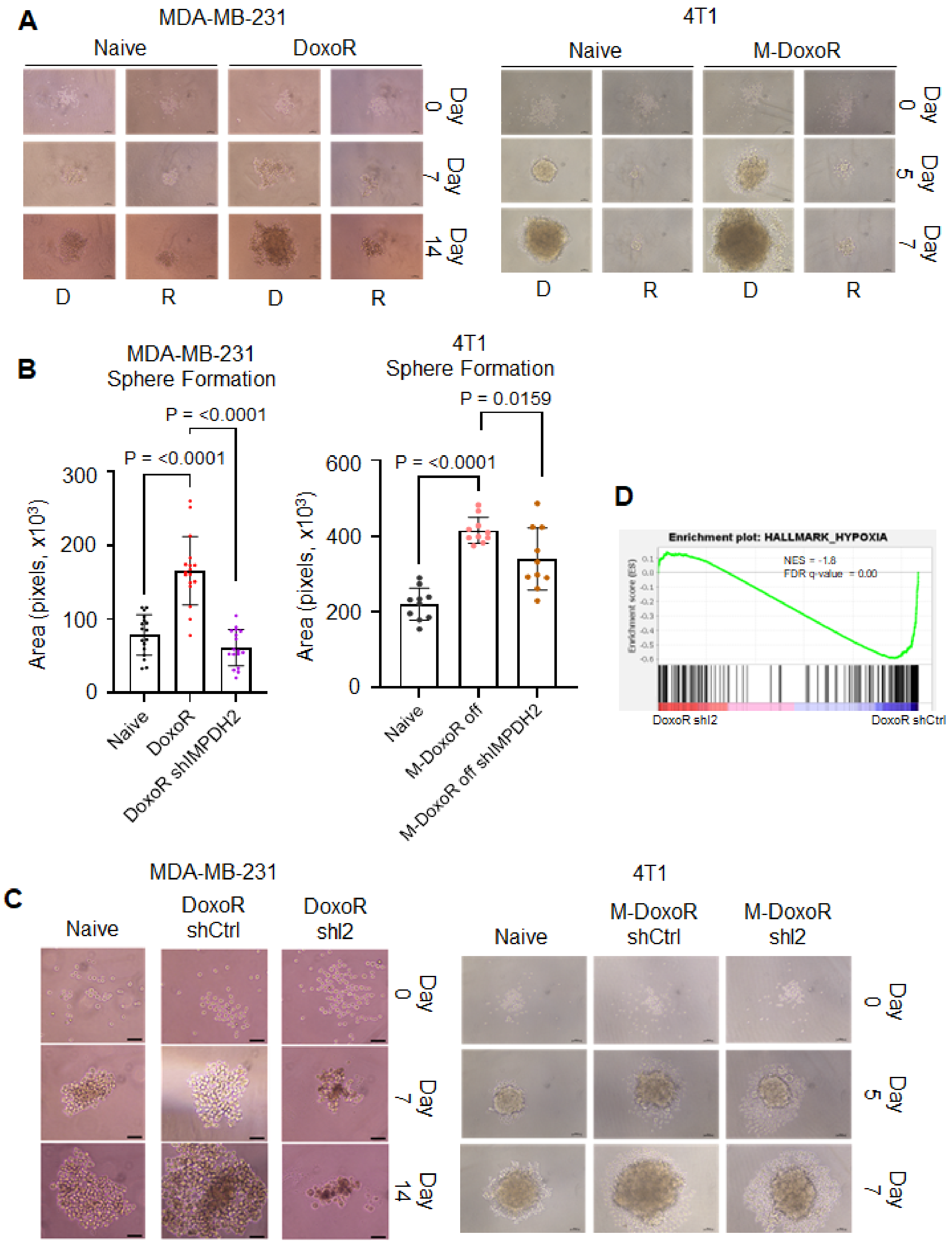

Supplemental Figure 5. IMPDH2 depletion suppresses tumorigenic phenotypes of DoxoR cells. (A) Representative images of tumor sphere formation in MDA-MB-231 (left) and 4T1 (right) naïve and resistant treated with DMSO (D, vehicle control) or ribavirin (R, 25μM). Scale bar, 100μm. **(B)** Tumor sphere formation analysis in MDA-MB-231 (left) and 4T1 (right) naïve and doxorubicin resistant transduced or not with shIMPDH2.

Data is average -/+ std dev of triplicates from 3 independent experiments. Statistics by two-tailed Student's *t*-test; ***p<0.001, ****p<0.0001. **(C)** Representative images of tumor sphere formation in MDA-MB-231 (left) and 4T1 (right) naïve and resistant transduced or not with shIMPDH2 (shI2). Scale bar, 100μm. **(D)** GSEA analysis for Hallmark_Hypoxia from RNA-seq of MDA-MB-231 DoxoR transduced or not with shIMPDH2 (shI2).

Supplementary Figure S6

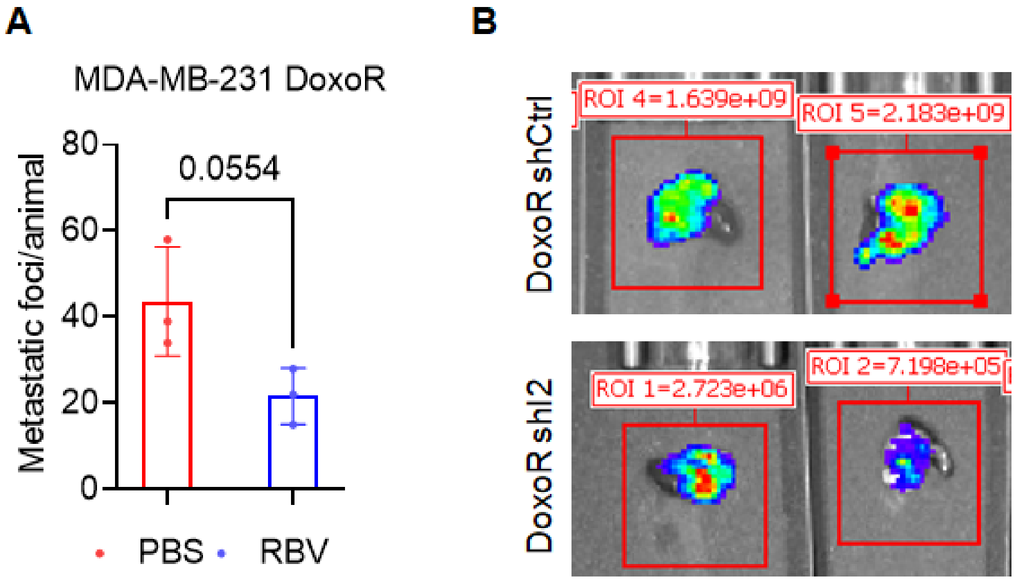

Supplemental Figure 6. IMPDH2 depletion suppresses metastasis *in vivo*. (A) 1x10^5 MDA-MB-231 DoxoR cells were injected in the tail vein of female NSG mice (n=3). Treatment with PBS or ribavirin (3mg/Kg in PBS) by i.p. injections (5days/week) started 4 days post-injection. Animals were humanely euthanized after 3 weeks. Experimental metastases were visualized with India ink stain and quantified manually. Statistics by two-tailed Student’s *t*-test. **(B)** Representative luciferase activity images of lungs of female NSG mice (n=5) injected with MDA-MB-231 DoxoR cells transduced with shIMPDH2 or corresponding non-silencing control and with a luciferase reporter construct as in Figure 5C.

Full unedited blot for Figure 2A

Blot

Ladder and membrane margins

Blot

Ladder and membrane margins

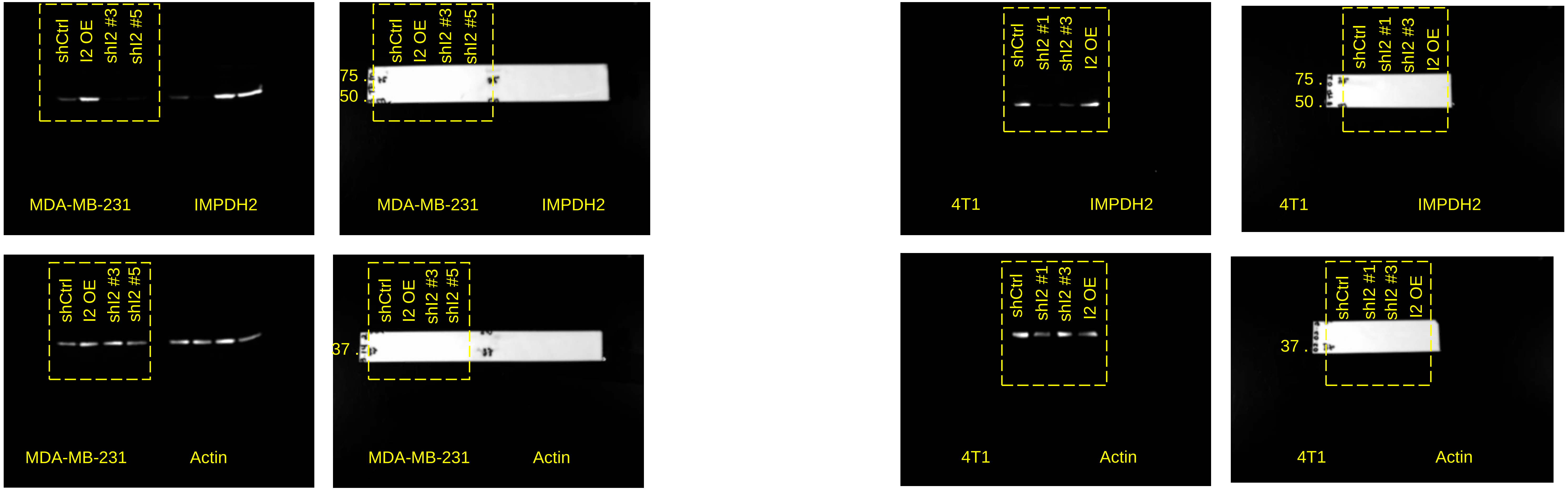

Full unedited blot for Figure 2F

Blot

Ladder and membrane margins

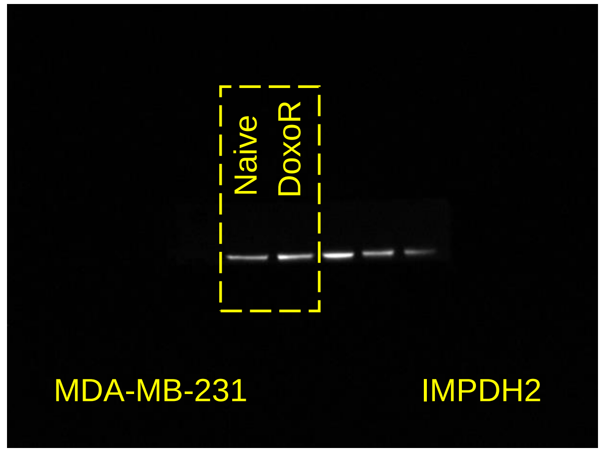

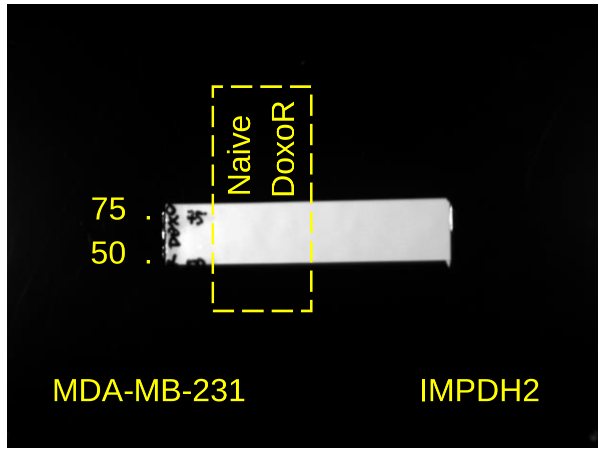

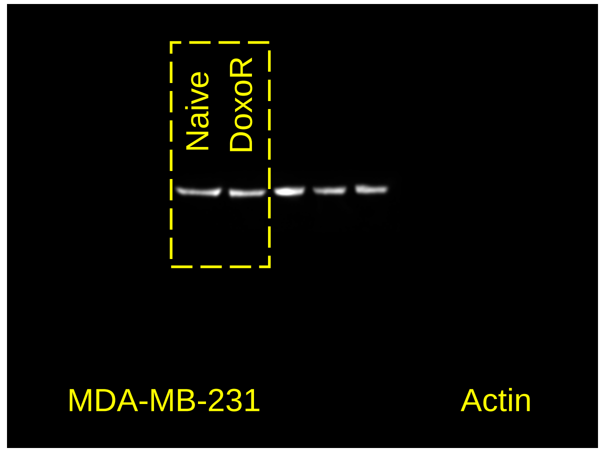

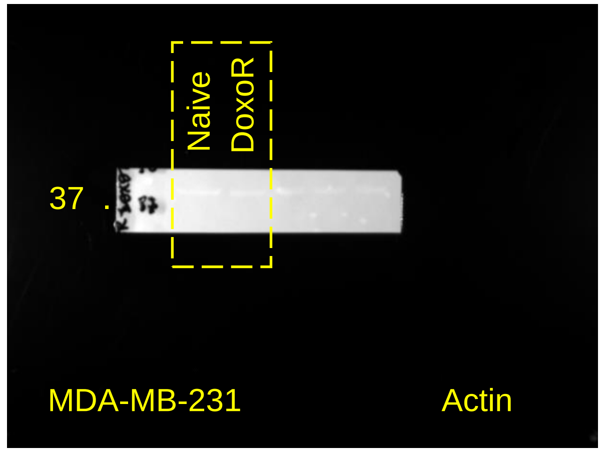

Full unedited blot for Figure 2G

Blot

Ladder and membrane margins

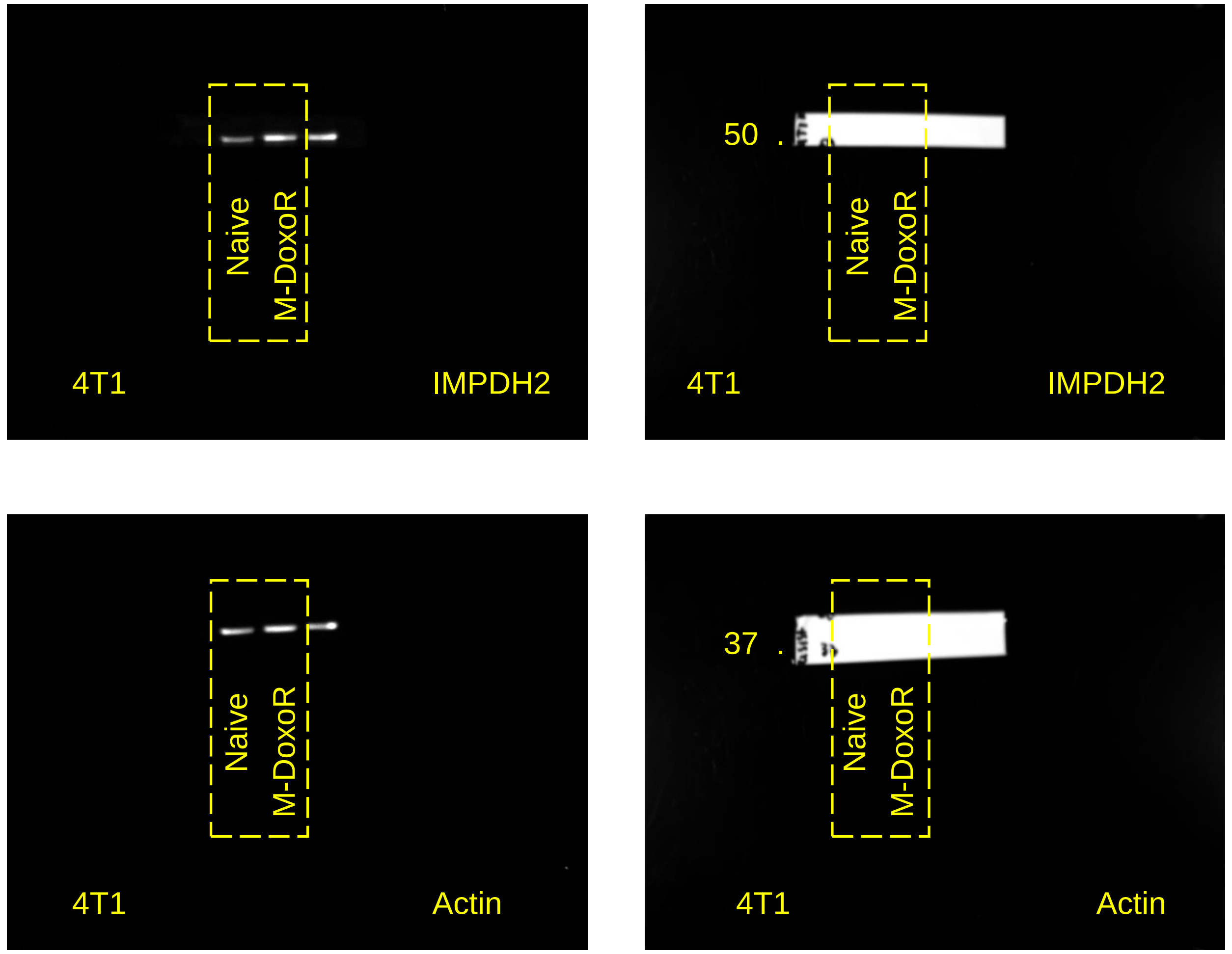

Full unedited blot for Figure 3C

Blot

Ladder and membrane margins

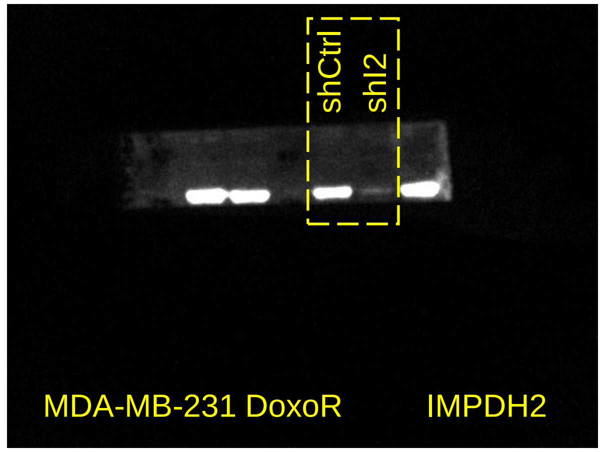

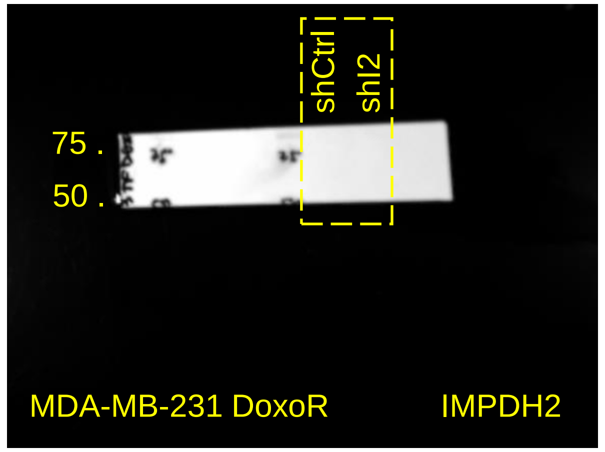

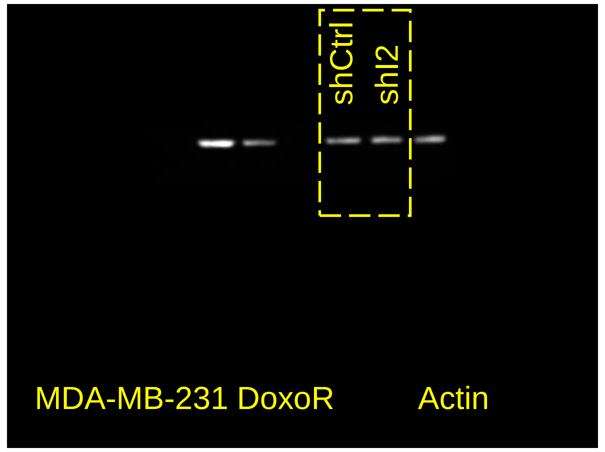

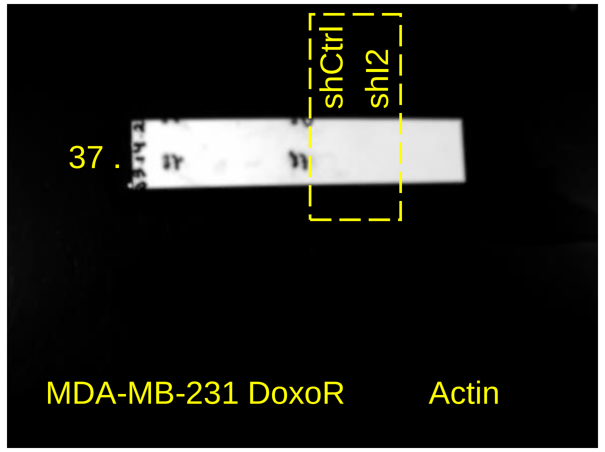

Full unedited blot for Figure 3D

Blot

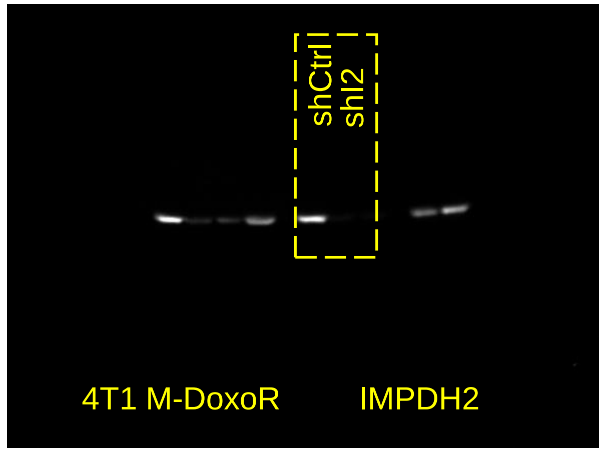

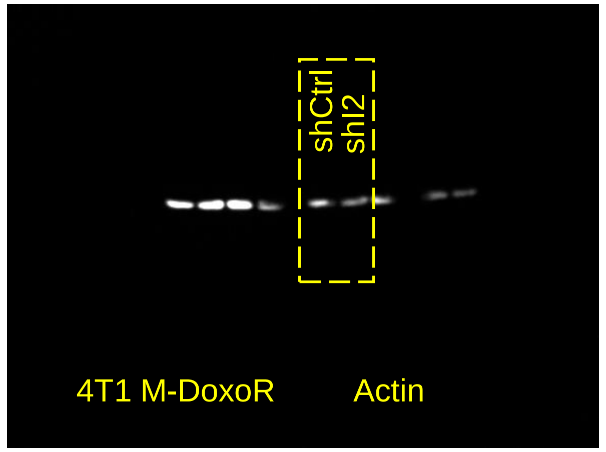

Ladder and membrane margins

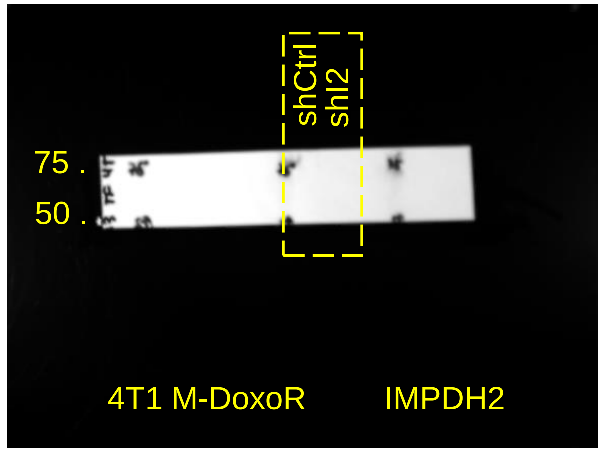

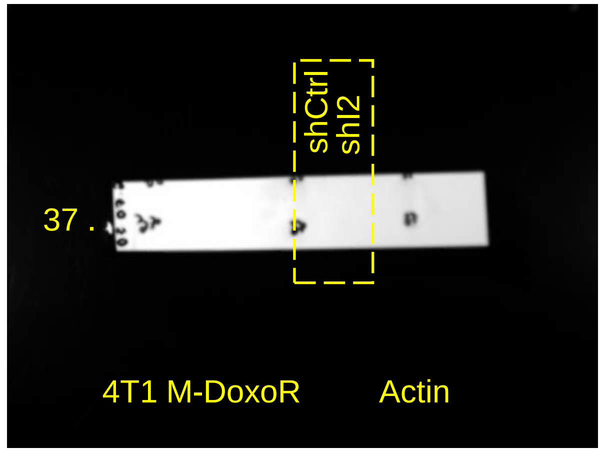

Full unedited blot for Supplemental Figure 2A

Blot

Ladder and membrane margins

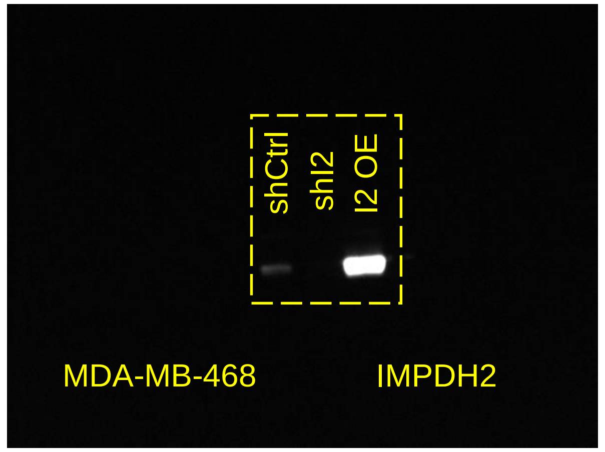

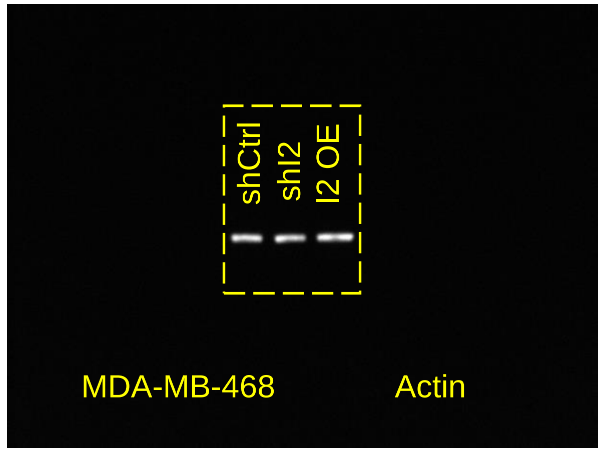

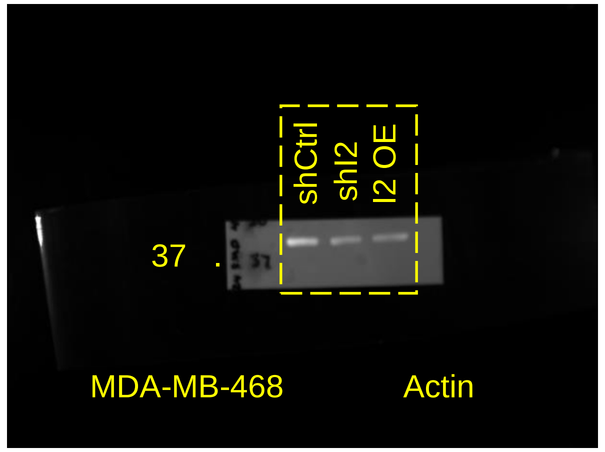

Full unedited blot for Supplemental Figure 2E

Blot

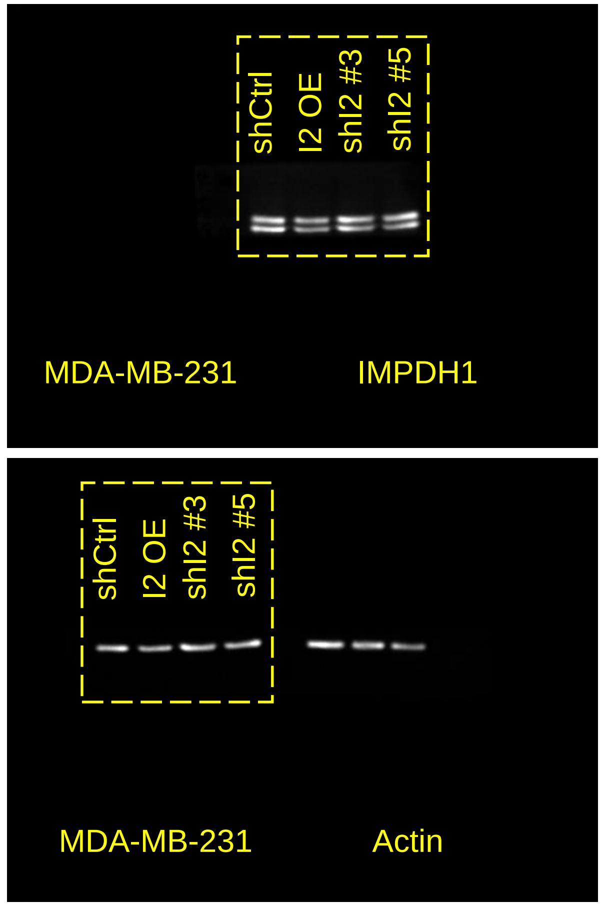

Ladder and membrane margins

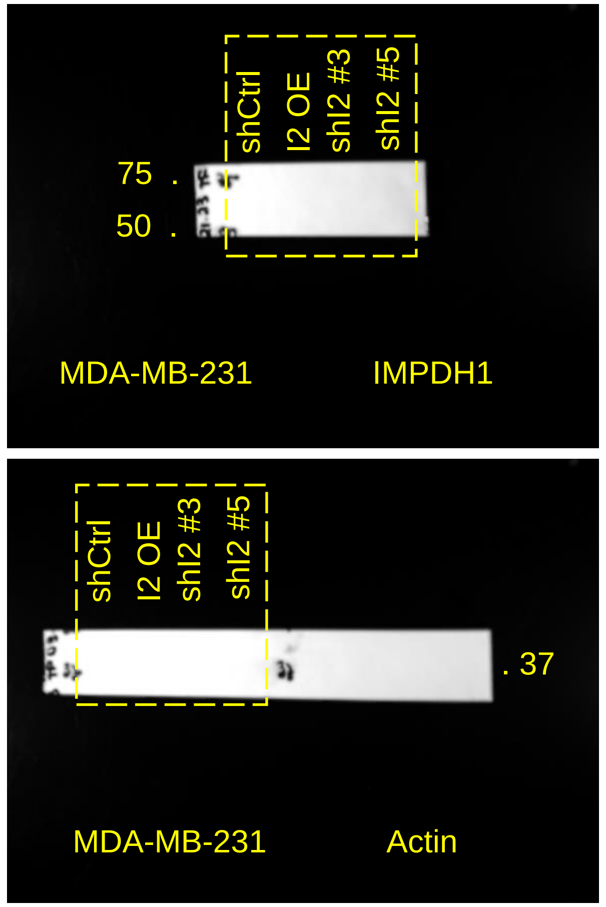

Blot

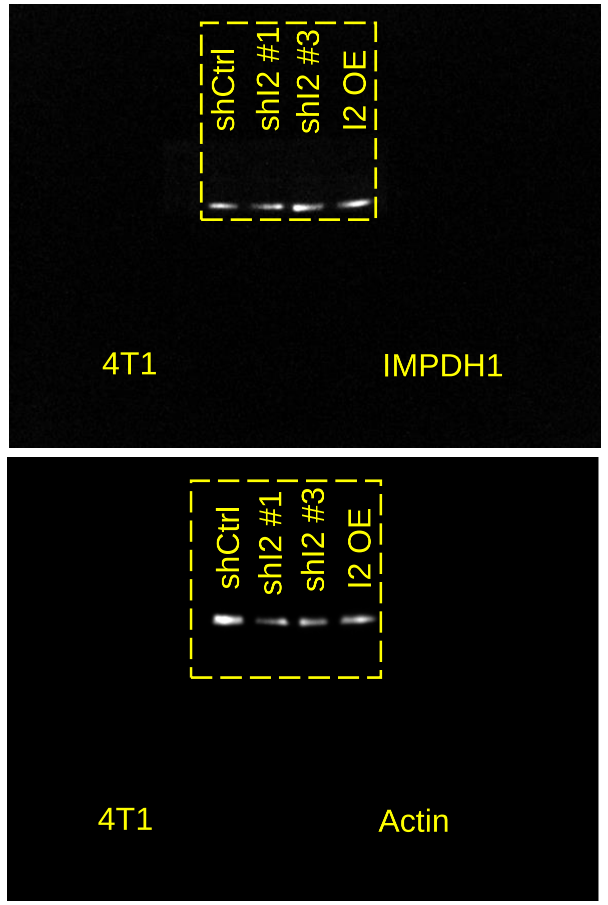

Ladder and membrane margins

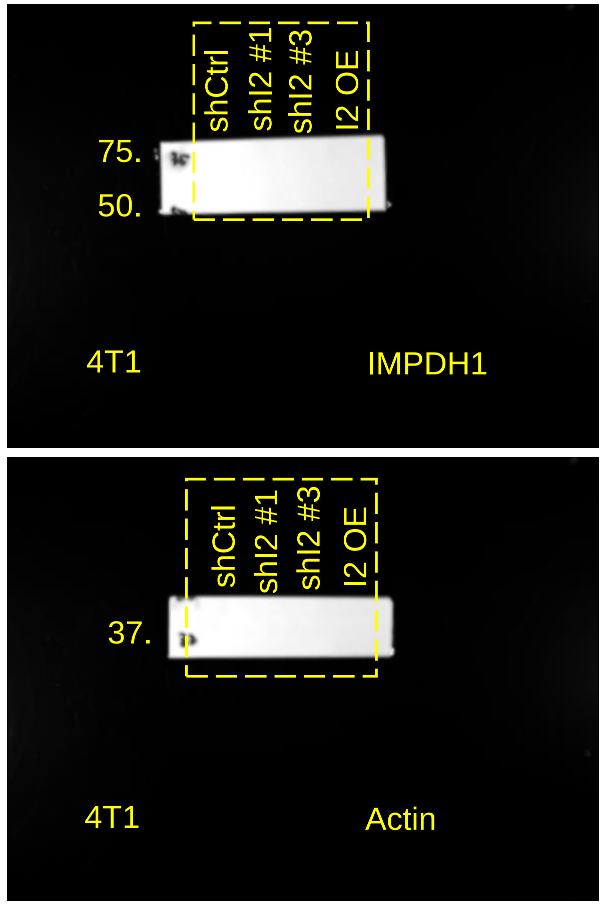

Blot

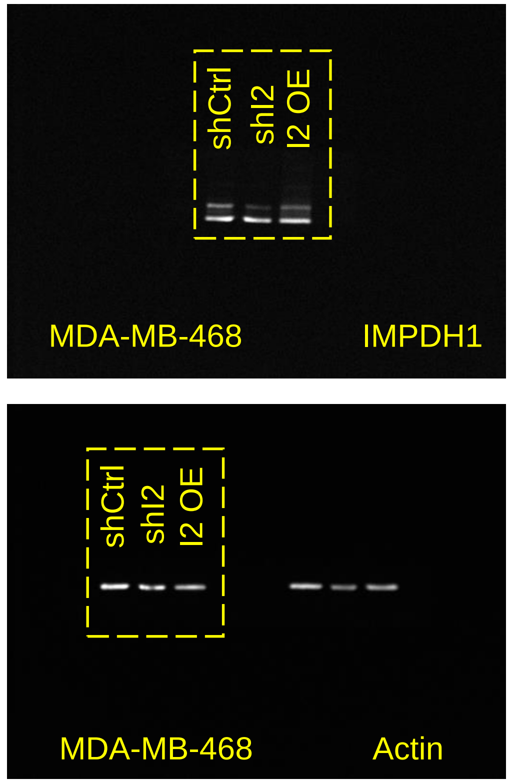

Ladder and membrane margins

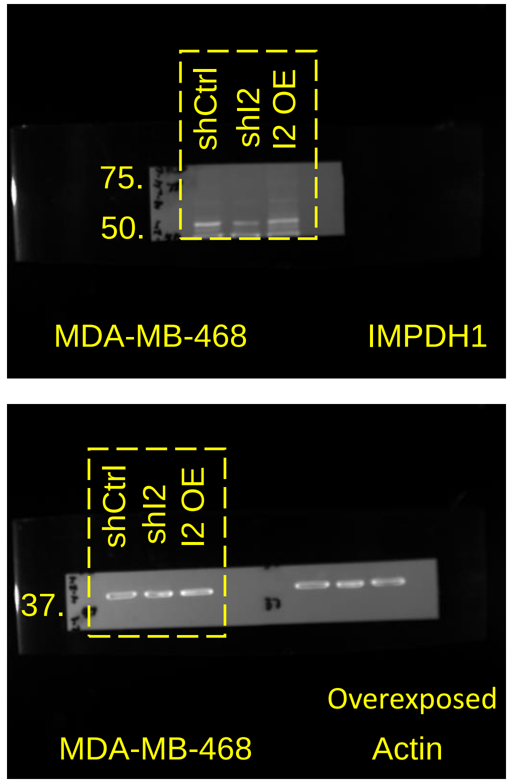

Full unedited blot for Supplemental Figure 2F

Blot | Ladder and membrane margins | Blot | Ladder and membrane margins

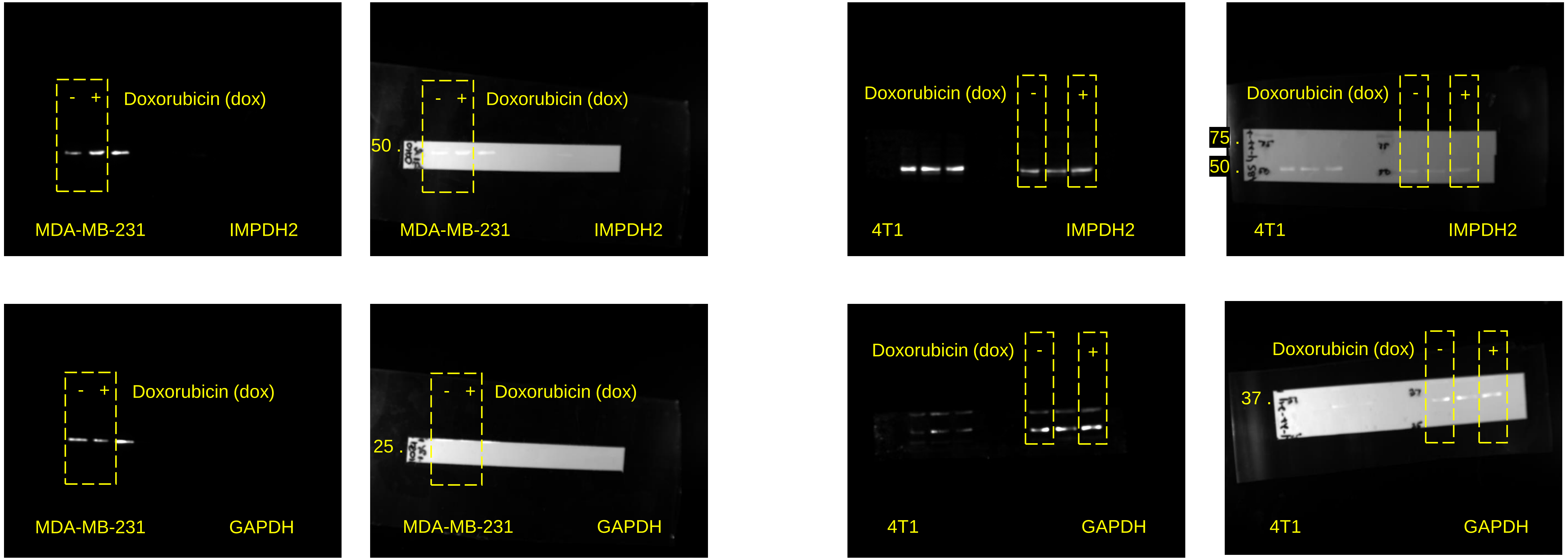

Full unedited blot for Supplemental Figure 4D

Blot

Ladder and membrane margins

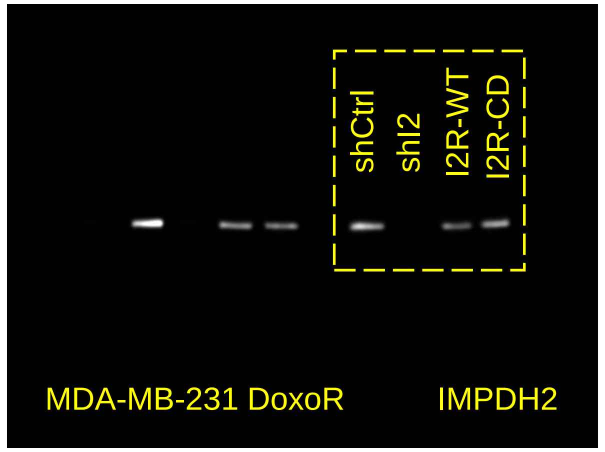

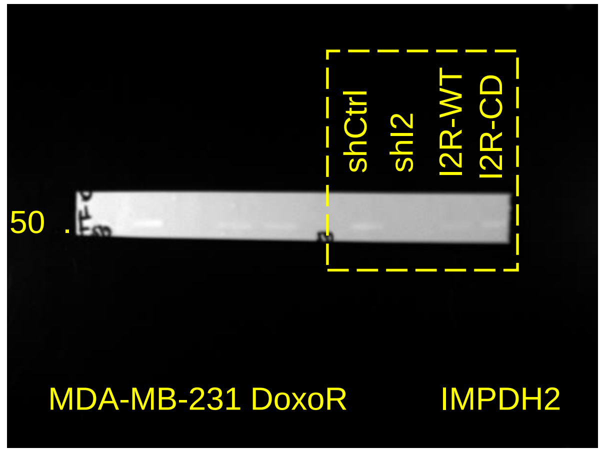

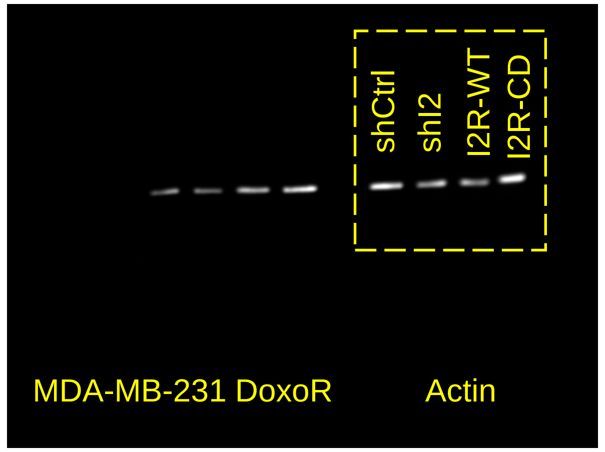

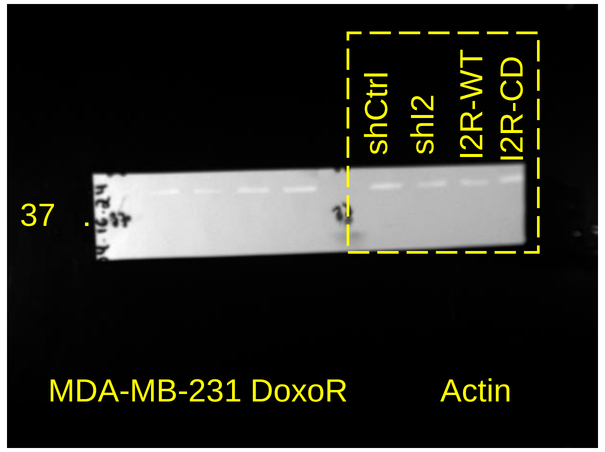

Full unedited blot for Supplemental Figure 4G

Blot

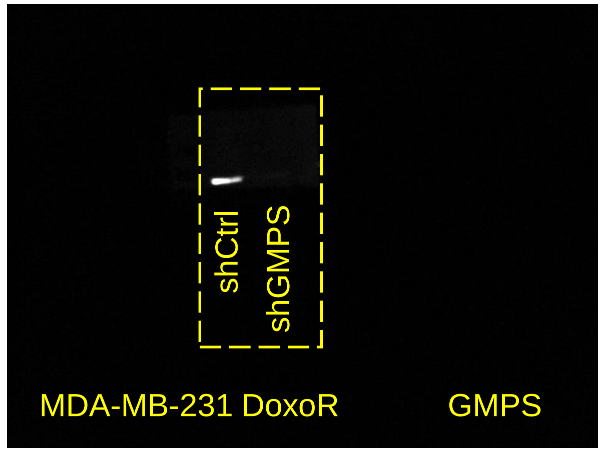

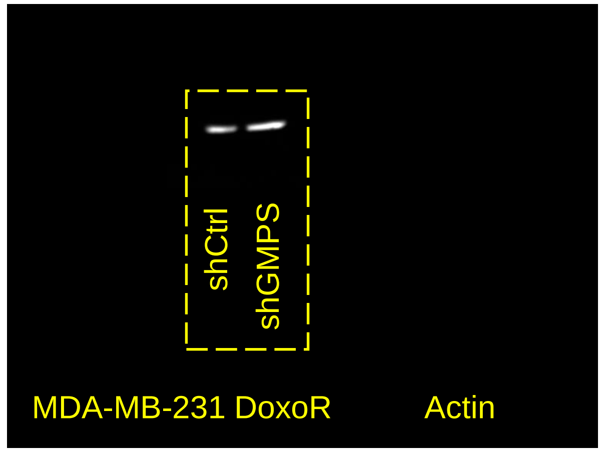

Ladder and membrane margins

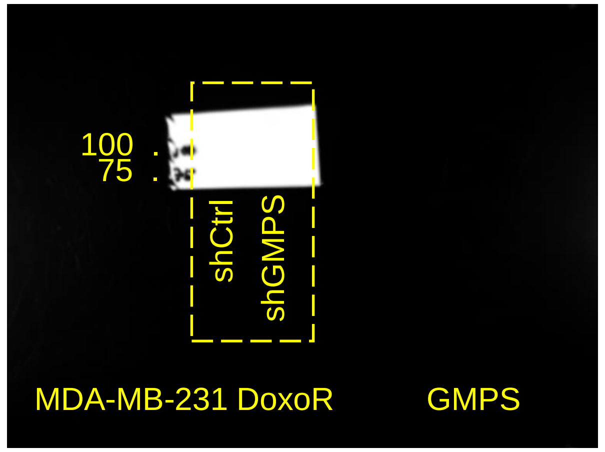

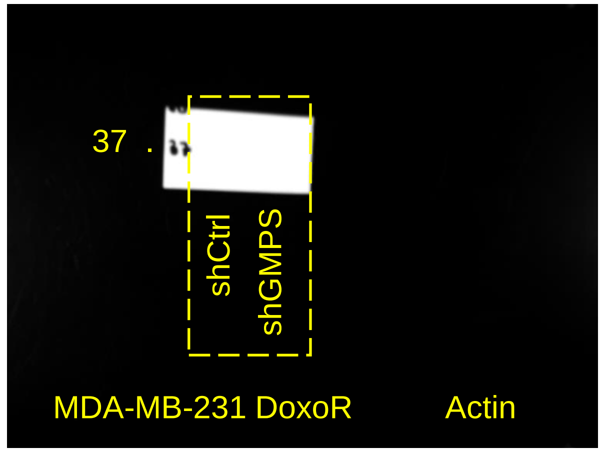

Blot

MDA-MB-231 DoxoR

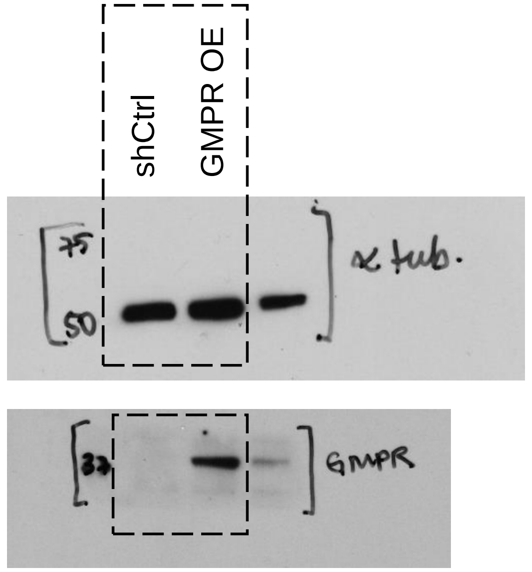

Supplement: Supplementary file 1 — Supplementary Information. [file 41598_2024_85094_MOESM1_ESM.pdf]
